# *Arabidopsis thaliana* RALF1 opposes brassinosteroid effects on root cell elongation and lateral root formation

**DOI:** 10.1093/jxb/eru099

**Published:** 2014-03-11

**Authors:** Tábata Bergonci, Bianca Ribeiro, Paulo H.O. Ceciliato, Juan Carlos Guerrero-Abad, Marcio C. Silva-Filho, Daniel S. Moura

**Affiliations:** ^1^Laboratório de Bioquímica de Proteínas, Departamento de Ciências Biológicas, Escola Superior de Agricultura Luiz de Queiroz, Universidade de São Paulo (ESALQ/USP), Piracicaba, SP, 13418–900, Brazil,; ^2^Laboratório de Biologia Molecular de Plantas, Departamento de Genética, Escola Superior de Agricultura Luiz de Queiroz, Universidade de São Paulo (ESALQ/USP), Piracicaba, SP, 13418–900, Brazil

**Keywords:** Root development, brassinolide, peptide hormone.

## Abstract

Rapid alkalinization factor (RALF) is a peptide signal that plays a basic role in cell biology and most likely regulates cell expansion. In this study, transgenic *Arabidopsis thaliana* lines with high and low levels of *AtRALF1* transcripts were used to investigate this peptide’s mechanism of action. Overexpression of the root-specific isoform *AtRALF1* resulted in reduced cell size. Conversely, *AtRALF1* silencing increased root length by increasing the size of root cells. AtRALF1-silenced plants also showed an increase in the number of lateral roots, whereas AtRALF1 overexpression produced the opposite effect. In addition, four AtRALF1-inducible genes were identified: two genes encoding proline-rich proteins (*AtPRP1* and *AtPRP3*), one encoding a hydroxyproline-rich glycoprotein (*AtHRPG2*), and one encoding a xyloglucan endotransglucosylase (*TCH4*). These genes were expressed in roots and involved in cell-wall rearrangement, and their induction was concentration dependent. Furthermore, AtRALF1-overexpressing plants were less sensitive to exogenous brassinolide (BL); upon BL treatment, the plants showed no increase in root length and a compromised increase in hypocotyl elongation. In addition, the treatment had no effect on the number of emerged lateral roots. AtRALF1 also induces two brassinosteroid (BR)-downregulated genes involved in the BR biosynthetic pathway: the cytochrome P450 monooxygenases *CONSTITUTIVE PHOTOMORPHISM AND DWARFISM* (*CPD*) and *DWARF4* (*DWF4*). Simultaneous treatment with both AtRALF1 and BL caused a reduction in AtRALF1-inducible gene expression levels, suggesting that these signals may compete for components shared by both pathways. Taken together, these results indicate an opposing effect of AtRALF1 and BL, and suggest that RALF’s mechanism of action could be to interfere with the BR signalling pathway.

## Summary

The peptide RALF regulates cell expansion via an as-yet-unknown mechanism. Here, we provide evidence that AtRALF1 may be negatively regulating cell expansion by interfering with the brassinosteroid signalling pathway.

## Introduction

Several physiological processes related to growth, development, defence, and reproduction are coordinated by intercellular communication. Peptide signals are involved in developmental processes and environmental responses in plants through the regulation of intercellular signalling (Ryan *et al.*, 2002; Boller, 2005; Matsubayashi and Sakagami, 2006; Moura and Silva-Filho, 2006).

Rapid alkalinization factor (RALF) is a peptide signal found throughout the plant kingdom that can exhibit either ubiquitous or tissue-specific expression patterns (Moura and Silva-Filho, 2006). The precursor of the RALF peptide is a preproprotein, indicating that this peptide is secreted (Pearce *et al.*, 2001); when Escobar *et al.* (2003) fused the tobacco RALF cDNA with that of green fluorescent protein, the resulting chimaeric protein was detected in both the endoplasmic reticulum and the apoplast. The RALF precursor is processed by convertases that target the dibasic site upstream of the active peptide, which is located at the C terminus of the precursor (Matos *et al.*, 2008; Srivastava *et al.*, 2009). A structure–activity study using the tomato RALF peptide showed that the motif ‘YISY’, which is located at positions 5–8 at the N terminus of the active peptide, is essential for activity (Pearce *et al.*, 2010).

Knowledge of the RALF signal transduction pathway is far from complete, and RALF-inducible genes are currently unknown. The rapid increase in the external pH of cell suspension cultures and the activation of a mitogen-activated protein kinase are among the earliest events in the pathway (Pearce *et al.*, 2001). Although the RALF receptor has not yet been identified, Scheer *et al.* (2005) identified two cell-surface SlRALF-binding proteins when studying the peptide in *Solanum lycopersicum* (SlRALF, previously LeRALF). AtRALF1, a root-specific isoform from *Arabidopsis thaliana*, causes a transient increase in the cytoplasmic Ca^2+^ concentration, suggesting that RALF peptides mediate a Ca^2+^-dependent signal transduction pathway (Haruta *et al.*, 2008).

A synthetic tomato RALF peptide was shown to inhibit root growth in tomato and *Arabidopsis* seedlings (Pearce *et al.*, 2001). SacRALF1, a peptide isolated from the leaves of the grass sugarcane, inhibits the formation of elongated cells in cell-suspension cultures, and SacRALF1 gene expression is observed in the elongating base of the leaves but not in the mature, non-elongating leaf tips (Mingossi *et al.*, 2010). When present in pollen germination medium, the SlRALF peptide inhibits the elongation of normal pollen tubes (Covey *et al.*, 2010), and the AtRALF1 isoform also inhibits hypocotyl elongation in dark-grown *Arabidopsis* seedlings (Mingossi *et al.*, 2010). In the most recent version of the *Arabidopsis* genome, 37 AtRALFs were identified (Lamesch *et al.*, 2011). The overexpression of two of these isoforms from *Arabidopsis*, AtRALF1 and AtRALF23, results in shorter and bushier *Arabidopsis* plants that displayed a characteristic semi-dwarf phenotype (Matos *et al.*, 2008; Srivastava *et al.*, 2009). When the single-copy RALF gene of the *Nicotiana attenuata* genome is silenced, the roots of the transgenic tobacco plants grow longer than those of the wild type (Wu *et al.*, 2007). Thus, all the evidence gathered thus far indicates that RALF peptides have a basic role in cell biology, and they most likely regulate cell expansion (Pearce *et al.*, 2001; Matos *et al.*, 2008; Srivastava *et al.*, 2009; Covey *et al.*, 2010; Mingossi *et al.*, 2010).

Cell expansion occurs through the loosening of the existing cell-wall architecture, which is accompanied by the synthesis of new wall components (Carpita and Gibeaut, 1993; McCann *et al.*, 2001; Cosgrove, 2005; Benatti *et al.*, 2012). The primary cell wall is a complex matrix of polysaccharides, structural proteins, and enzymes (Carpita and Gibeaut, 1993; Fry, 2004). Hydroxyproline-rich glycoproteins (HRGPs) and proline-rich proteins (PRPs) are structural proteins that may be involved in cell-wall expansion (Showalter, 1993; Kieliszewski and Lamport, 1994; Cosgrove, 2005). Cell-wall enzymes such as expansin and xyloglucan endotransglucosylase (XET) are also related to cell-wall expansion due to their promotion of wall loosening (Fry *et al.*, 1992; Cosgrove, 1999; Eklöf and Brumer, 2012). The mechanisms involved in cell expansion are regulated by brassinosteroid (BR), auxin, ethylene, gibberellin, and cytokinin signalling (Smalle *et al.*, 1997; Wang *et al.*, 2002; Fu and Harberd, 2003; Parry and Estelle, 2006; Vanstraelen and Benková, 2012).

BRs such as brassinolide (BL) are involved in hypocotyl elongation and root growth (Clouse *et al.*, 1996; Clouse and Sasse, 1998; Müssig *et al.*, 2003). BR-mediated cell expansion involves wall-modifying proteins, such as XETs, as well as cortical microtubule reorientation (Mayumi and Shibaoka, 1995; Clouse and Sasse, 1998; Wang *et al.*, 2012). The activity of BL is mediated by a leucine-rich repeat receptor kinase called BRI1 (Clouse *et al.*, 1996; He *et al.*, 2000). BR binding to BRI1 triggers the autophosphorylation of the kinase domain and the subsequent recruitment of the co-receptor BRI1-associated receptor kinase (BAK1), resulting in the activation of BR-responsive genes (Clouse, 2004; Vert *et al.*, 2005; Chinchilla *et al.*, 2009; Li and Jin, 2007).

Little is known about the cross-talk between RALF peptides and other plant signals. In poplar, the *PtdRALF2* gene is downregulated after methyl jasmonate treatment, whereas auxin and cytokinin treatments do not affect *PtdRALF2* RNA levels (Haruta and Constabel, 2003). The *Arabidopsis* gene *AtRALF23* is downregulated upon treatment with BRs, and plants that overexpress the *AtRALF23* gene exhibit compromised BL-induced hypocotyl elongation (Srivastava *et al.*, 2009).

In this study, we showed that silencing of the *AtRALF1* gene in *Arabidopsis* plants increased cell elongation and the formation of lateral roots, whereas *AtRALF1* overexpression showed the opposite effects. To track the effects of the peptide at the molecular level, we identified and report for the first time six RALF-inducible genes. Four of these genes are related to cell-wall rearrangement and two were characterized previously as BR-downregulated genes involved in the BR biosynthetic pathway. In addition, we showed that AtRALF1-overexpressing plants have a compromised response to exogenously applied BR. Our data suggest that AtRALF1 opposes the action of BR in *Arabidopsis* plants and that the interplay between these two signals might collaborate in the regulation of cell expansion.

## Materials and methods

### Plant materials and growth conditions

*Arabidopsis* plants that overexpress *AtRALF1* have been described previously (Matos *et al.*, 2008). Seeds for the BR-insensitive mutant *bri1* (At4g39400, CS3723) were obtained from the *Arabidopsis* Biological Resource Center, Ohio State University, Columbus, USA. For all seedling experiments, *Arabidopsis* seeds (ecotype Col-0) were surface sterilized and cold treated (4 °C) for 4 d in the dark. Seedlings were grown in soil or in half-strength medium containing Murashige and Skoog (MS) salts without vitamins and sucrose (PhytoTechnology Laboratories), KOH-adjusted pH 5.8, and containing 0.9% (w/v) of Gellan Gum Powder (PhytoTechnology Laboratories) in a growth room at 22±2 °C with a light regime of 16h light and 8h dark.

### Plasmid construction

The *AtRALF1* coding region was amplified from *Arabidopsis* genomic DNA using standard PCR with the primers AtRALF1F and AtRALF1R (Supplementary Table S1 available at *JXB* online). The interfering RNA construct used to silence the *AtRALF1* gene (irAtRALF1) was generated using Gateway technology (Invitrogen) and the vectors pENTR/D-TOPO (Invitrogen) and pk7GWIWG2I (Karimi *et al.*, 2002) following the manufacturer’s instructions. All constructs were verified by DNA sequencing.

### Plant transformation

Plants were inoculated with the *Agrobacterium tumefaciens* strain GV3101 carrying the pk7GWIWG2I vector using the floral dip method (Clough and Bent, 1998). Seeds from transformed plants were plated in half-strength medium containing MS salts without vitamins and sucrose and 100mg l^–1^ of kanamycin (Sigma). Transgenic homozygous *Arabidopsis* lines from the T3 generation were used in all experiments.

### Hormone treatments

Seedlings were grown on vertical plates containing half-strength medium containing MS salts without vitamins and sucrose with different concentrations of 24 epi-brassinolide (BL; PhytoTechnology Laboratories) or indole-3-acetic acid (IAA; PhytoTechnology Laboratories). BL and IAA were prepared as 2mM stock solutions; IAA was dissolved in 10M potassium hydroxide and then diluted in water. The BL stock solution was prepared in 10% (w/v) ethanol. Further dilutions were made in water, and the final concentration of the solvents used was applied in the controls and the experimental conditions. Seedlings were photographed using a Nikon (CoolPix S202) digital camera 10 d after germination, and primary root lengths, hypocotyl elongation, rosettes, and leaves were measured using ImageJ (National Institutes of Health, USA). The number of emerged lateral roots (>1mm) was also recorded 10 d after germination. All experiments were repeated at least three times (independent biological replicates).

Hypocotyl measurements were taken as described by Weigel and Glazebrook, (2002). After cold treatment (4 d at 4 °C), the seeds were placed in half-strength liquid medium containing MS salts without vitamins and sucrose, and incubated with gentle agitation on a rotary shaker in the growth room. Seeds were exposed to light for 1h and were then kept in the dark (for dark experiments) or maintained in the growth room with 16h light and 8h dark (for light experiments). The recombinant AtRALF1 peptide (_His_AtRALF1) and BL were added 1 d after germination, and measurements were taken 6 d after treatment.

For semi-quantitative reverse transcription (RT)-PCR and quantitative (q)RT-PCR analysis, 10-d-old seedlings were placed in half-strength liquid medium containing MS salts without vitamins and sucrose, and incubated with gentle agitation on a rotary shaker in the growth room. Different concentrations of _His_AtRALF1 and/or BL were then added to the medium.

### Production and purification of recombinant _His_AtRALF1

The AtRALF1 coding region was amplified using standard PCR with genomic DNA as the template and specific primers for the correction of rare codons [primers A (forward) and C (reverse); Supplementary Table S1 available at *JXB* online]. The final amplification of the insert was performed using specific primers [primers D (forward) and C (reverse); Supplementary Table S1 available at *JXB* online]. The amplified fragment was fused to the C terminus of a 6×His tag using the pET28b expression vector (Novagen) and introduced into *Escherichia coli* strain BL21. Cells harbouring the plasmids were grown at 37 °C at 250rpm until they reached an optical density at 600nm of 0.7 and then were treated with isopropyl β-d-1-thiogalactopyranoside (1mM) for 4h to induce protein expression. Bacterial cells were harvested by centrifugation and resuspended in denaturing buffer [100mM NaH_2_PO_4_, 10mM Tris/HCl (pH 8.0), 8M urea] and lysed using cell disruption by nitrogen decompression in a Parr bomb. The lysate was centrifuged at 16 000*g* for 40min at room temperature. The supernatant was then applied to an affinity chromatography column containing Ni-NTA resin (Qiagen) to purify the peptide. The purified peptide was lyophilized, purified, and quantified by high-performance liquid chromatography using a C18 reversed-phase column (Kromasil) as described previously (Pearce *et al.*, 2001).

### Semi-quantitative RT-PCR and qRT-PCR analysis

Total RNA was isolated from the roots of *Arabidopsis* plants using Trizol reagent according to the manufacturer’s instructions (Invitrogen) and treated with DNAse I (Invitrogen). cDNA was synthesized using 1 µg of RNA and the ImProm-II Reverse Transcription System (Promega). For semi-quantitative RT-PCR analyses, an aliquot of cDNA was used as template in the PCR, which was performed for 27 cycles, unless indicated otherwise, using gene-specific primers (Supplementary Table S1 available at *JXB* online). The glyceraldehyde 3-phosphate dehydrogenase (*GAPDH*) gene was used as a reference to show the equal loading of cDNA in the reactions. All the experiments were performed at least three times (independent biological replicates), and a representative experimental result is shown. Evaluation of RT-PCR signals via densitometry after normalization to the housekeeping gene *GAPDH* was made using ImageJ.

qRT-PCR was performed using 20-fold-diluted cDNA and the Maxima SyBR Green Rox/qPCR Master Mix (Thermo Scientific) on a StepOne™ Real-Time PCR System (Applied Biosystems). The primers used are listed in Supplementary Table S1 (available at *JXB* online). The *GAPDH* gene was used as an internal control. Three replicates were analysed for each biological sample along with a template-free reaction as a negative control. The threshold cycle (*C*_T_) was determined automatically by the instrument, and the fold change in each gene was calculated using the equation 2^–∆∆*C*T^ (Livak and Schmittgen, 2001). An arbitrary value of 1 was attributed to control treatments and wild-type plants. Graphs shown are from one representative biological replicate.

### Measurements of cell length

Seedlings were grown vertically on plates containing half-strength MS salts without vitamins and sucrose for root measurements. For hypocotyl measurements, unless described otherwise, seedlings were kept in the dark in plates containing half-strength liquid medium containing MS salts without vitamins and sucrose. Measurements were taken 10 d after germination in epidermal cells located at the base of the hypocotyls. For propidium iodide staining of the cell wall, the growth medium was replaced with a 1mg ml^–1^ propidium iodide solution (Sigma), and the cells were imaged 15min after the addition of the stain. Cells in the endodermis of the root differentiation zone (presence of root hairs), located at approximately 800 µm from the tip, were visualized and measured using confocal microscopy (Olympus FV1000). At least 30 cells per root and six cells per hypocotyl were analysed. Fifteen plants frrom each genetic background were used for root and hypocotyl cell measurements. The wavelengths for excitation and emission were 555 and 655nm, respectively. Image processing was completed using Olympus FluoView software.

### Statistical analyses

For statistical analyses, the Infostat Statistics Base software package (version 2012e; Córdoba, Argentina) was used. Means were compared using the Tukey HSD (honestly significant difference) test in conjunction with analysis of variance (Steel *et al.*, 1996).

## Results and discussion

### Reduction of *AtRALF1* transcripts in irAtRALF1 plants increases root length, lateral root number, hypocotyl elongation, and cell length

**Fig. 1.**
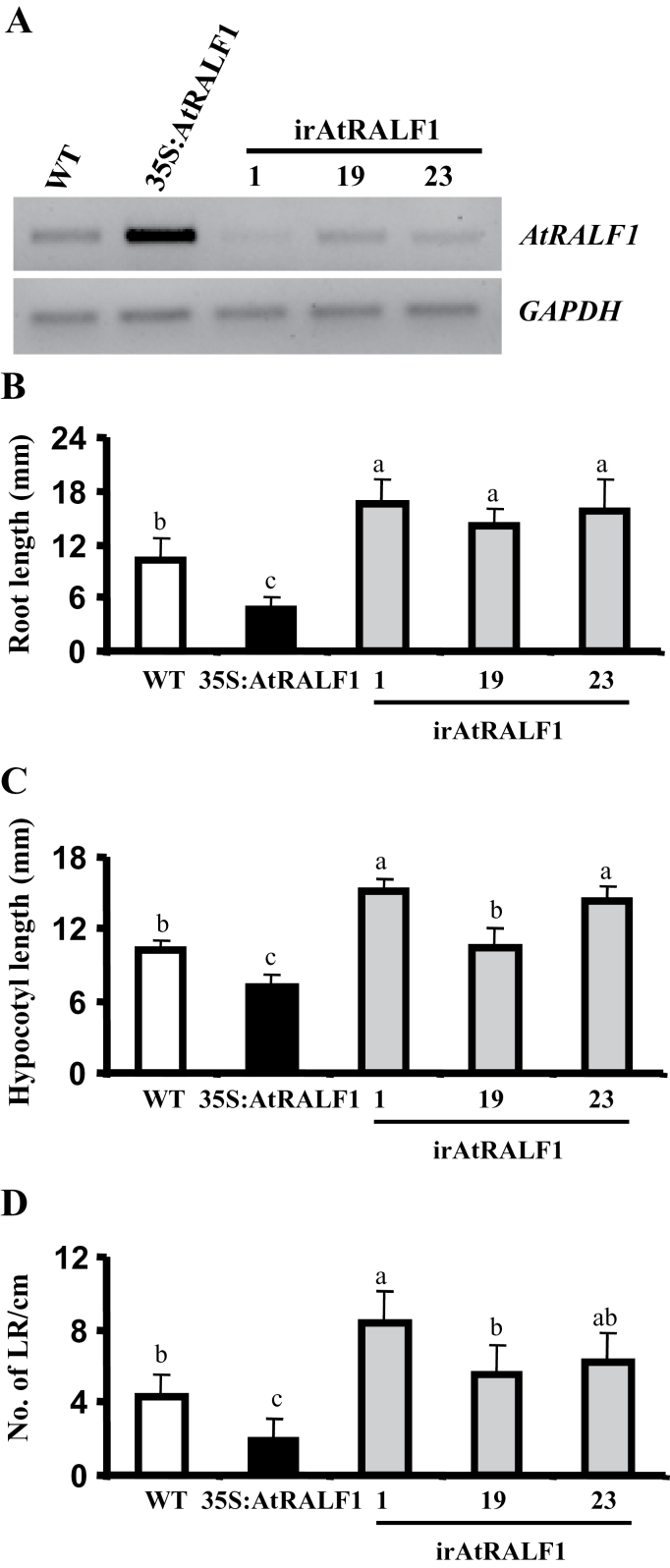
*AtRALF1* gene silencing in transgenic lines (irAtRALF1). (A) Transcript levels of the *AtRALF1* gene in transgenic plants. Transcript levels were examined by RT-PCR using RNA samples extracted from 10-d-old roots of plants grown on half-strength MS agar plates. *GAPDH* was used as an internal control. WT, wild type. (B) Root length of *AtRALF1*-overexpressing (35S:AtRALF1, black columns) and *AtRALF1*-silenced (irAtRALF1, grey columns) transgenic lines. (C) Hypocotyl length of etiolated transgenic plants. (D) Number of emerged lateral roots per cm in transgenic plants. Root length and number of emerged lateral roots were measured and counted in 5- and 10-d-old seedlings respectively (*n*>30). Hypocotyl length was measured in 5-d-old etiolated plants. Error bars indicate standard deviation (SD). Columns with the same letter are not significantly different (*P*<0.01). All experiments were repeated at least three times (independent biological replicates).

An interfering RNA construct (irAtRALF1) was used to silence the *AtRALF1* gene and to investigate the effects of the lack of AtRALF1 peptide in *Arabidopsis* plants. Thirty lines were obtained, and three lines with different levels of *AtRALF1* transcripts were selected for the evaluation of root growth, hypocotyl elongation, and lateral root formation. The *AtRALF1* transcript levels in the roots of selected irAtRALF1 lines 1, 19, and 23 were lower, similar, and slightly lower, respectively, when compared with wild type (Fig. 1A). Ratios of the AtRALF1 band intensities in the transgenic plants versus the control wild-type plants are shown in Supplementary Table S2 available at *JXB* online. A plant line that overexpresses *AtRALF1* (35S:AtRALF1) was obtained previously (Matos *et al.*, 2008) and is shown for comparison. irAtRALF1 plants showed an average 42.8% increase in root length (Fig. 1B), whereas, in 35S:AtRALF1 plants, root length was observed to decrease by a similar percentage. The increase in root length observed in irAtRALF1 plants was proportional to the level of *AtRALF1* transcripts detected by RT-PCR; the lower the transcript level, the longer the roots. Similar results were reported by Wu *et al.* (2007) in *N. attenuata* plants bearing a construct to silence the only *N. attenuata* RALF isoform. Hypocotyl elongation in dark-grown *Arabidopsis* plants is due to an increase in cell length and not to cell division (Gendreau *et al.*, 1997). Hypocotyl elongation was compromised in the 35S:AtRALF1 plants, and irAtRALF1 lines 1 and 23 showed longer hypocotyls compared with controls (Fig. 1C). Again, the increase in hypocotyl length in irAtRALF1 plants, approximately 33% in irAtRALF1 lines 1 and 23, was proportional to the decrease observed in 35S:AtRALF1 plants, which was approximately 30%. The irAtRALF1-19 line showed normal hypocotyl elongation in spite of the reduced levels of *AtRALF1* transcripts. Because the irAtRALF1-19 line had the least reduction in transcript levels, we believe that the reduction in this line is not sufficient to cause a visible effect on the hypocotyls. The irAtRALF1-1 line showed the lowest levels of *AtRALF1* transcripts and was used for further experiments. The wild-type phenotype was recovered in irAtRALF1 plants upon exogenous treatment with the _His_AtRALF1 peptide (Supplementary Fig. S1 available at *JXB* online).

In *Arabidopsis*, the lateral roots are derived from pericycle founder cells located opposite xylem poles (Blakely *et al.*, 1982; Malamy and Benfey, 1997; Péret *et al.*, 2009). Auxin, cytokinin, BRs, and ethylene act to control lateral root formation and emergence (Casimiro *et al.*, 2001; Bao *et al.*, 2004; Laplaze *et al.*, 2007; Negi *et al.*, 2008). Cytokinin and ethylene act as auxin antagonists (Laplaze *et al.*, 2007; Lewis *et al.*, 2011), whereas auxin and BRs act synergistically (Bao *et al.*, 2004). Interestingly, the number of emerged lateral roots in 35S:AtRALF1 plants was lower than in wild-type plants (average reduction of 60%), whereas the opposite occurred in irAtRALF1 (average increase of 60%) (Fig. 1D). These data indicated the possible interference of the peptide with root architecture maintenance. Recently, overexpression of the *AtRALF8* gene in *Arabidopsis* also caused a reduction in the number of lateral roots (Atkinson *et al.*, 2013).

**Fig. 2.**
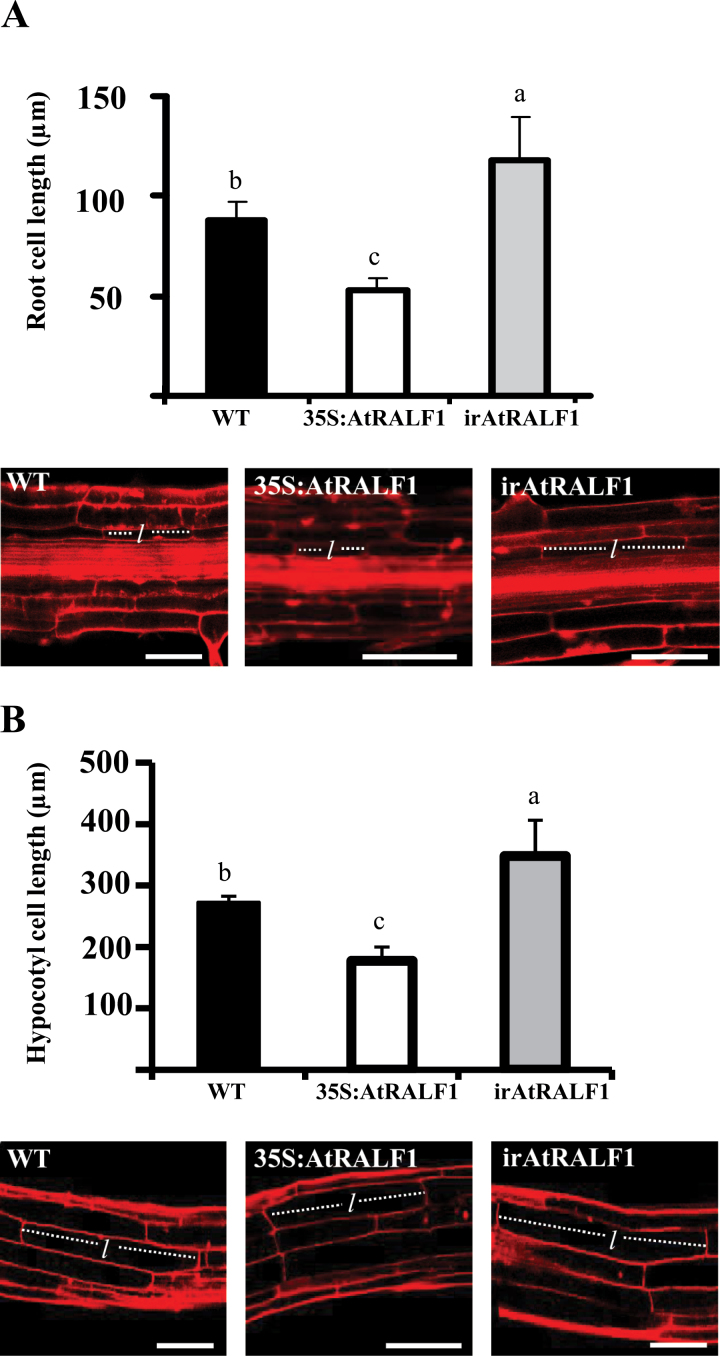
Root and hypocotyl cell length of *AtRALF1*-overexpressing (35S:AtRALF1) and *AtRALF1*-silenced (irAtRALF1) transgenic lines. (A) Root cell length of 35S:AtRALF1 plants (black columns) and irAtRALF1 (grey columns) plants. Panels below the graph are confocal images of the root differentiation zone. Cells from root endodermis of 10-d-old seedlings were measured (*n*=30 cells per root). WT, wild type. (B) Hypocotyl cell length of 35S:AtRALF1 (black columns) and irAtRALF1 (grey columns) plants. Panels below the graph are confocal images of the base of hypocotyls. Epidermal cells from hypocotyls of 10-d-old seedlings were measured (*n*=6 cells per hypocotyl). Error bars indicate SD. Columns followed by the same letter are not significantly different (*P*<0.01). Bars, 100 µm. The length of representative cells is indicated by ‘*l*’. (This figure is available in colour at *JXB* online.)

To prove that the increase in root length and hypocotyl elongation was due to an increase in cell length, we measured the lengths of root and hypocotyl cells in irAtRALF1 and 35S:AtRALF1 plants and compared these with cells from wild-type plants (Fig. 2A, B). Cells from the endodermis of the differentiation zone of the roots of 35S:AtRALF1 plants were 44% smaller than wild-type root cells in the same root zone, and root cells from irAtRALF1 plants were 30% larger than wild-type cells also from the differentiation zone. The endodermis layer was chosen for measurements due to the high levels of endogenous *AtRALF1* gene expression predicted by *in silico* data (eFP-browser, Birnbaum *et al.*, 2003; Winter *et al.*, 2007). Hypocotyl measurements performed on epidermal cells along the axis of elongation showed that 35S:AtRALF1 cells were 34% shorter than in the wild type, and irAtRALF1 cells were 27.6% longer than the hypocotyl cells from wild-type plants. Although *AtRALF1* is expressed predominantly in roots (Haruta *et al.*, 2008), its partial silencing also produced larger leaves and rosettes in irAtRALF1 plants (Supplementary Fig. S2 available at *JXB* online). Whilst we did not investigate these effects, the large rosettes and leaves could be due to the unintentional silencing of another RALF isoform present in the *Arabidopsis* genome. We evaluated the gene expression of seven RALF isoforms, including those closest in sequence to *AtRALF1* (*AtRALF22*, -*23*, and -*33*), and did not observe any significant reduction in their mRNA levels (Supplementary Fig. S3 available at *JXB* online). The results showed that the reduced root growth and hypocotyl elongation observed in 35S:AtRALF1 plants were a consequence of reduced cell size and that partial silencing of the *AtRALF1* gene led to an increase in cell size. Our data support a role for AtRALF1 in cell expansion through the inhibition of cell elongation.

### AtRALF1 induces the expression of genes involved in cell-wall rearrangement

**Fig. 3.**
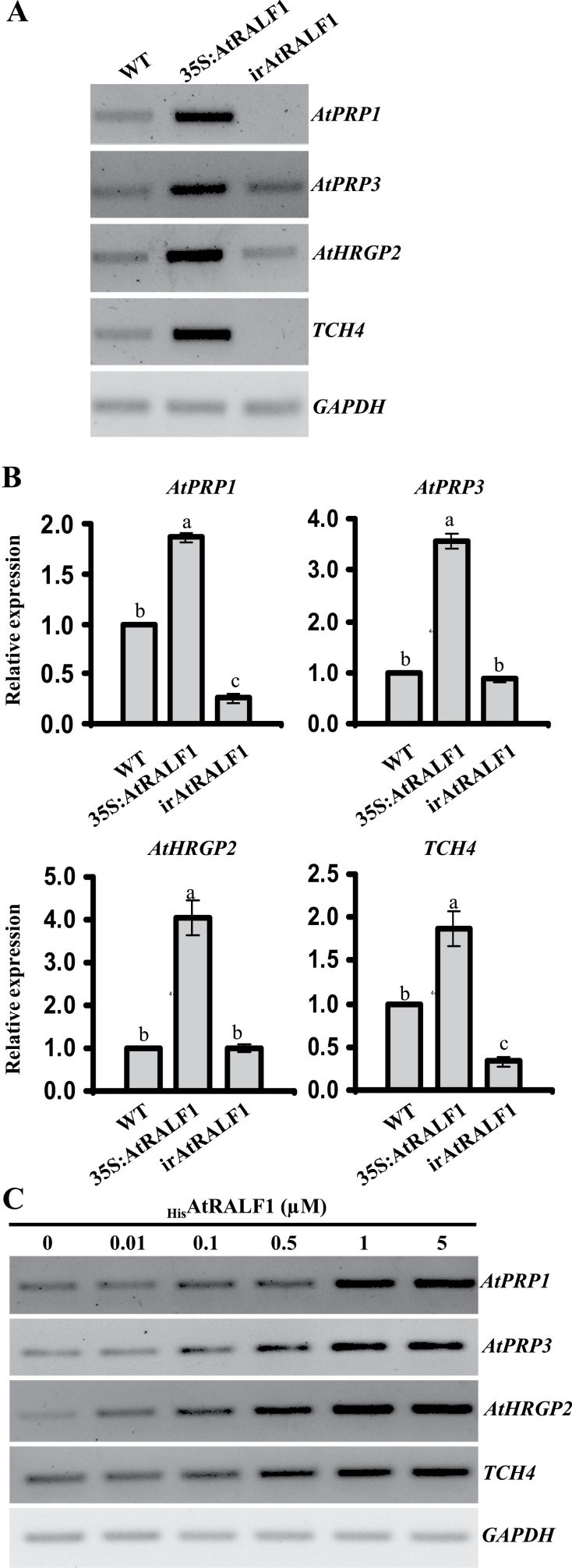
AtRALF1-inducible genes. (A, B) Semi-quantitative RT-PCR (A) and qRT-PCR gene expression analyses (B) performed in roots of 10-d-old *AtRALF1*-overexpressing (35S:AtRALF1), *AtRALF1*-silenced (irAtRALF1) transgenic lines and wild-type (WT) plants. Error bars indicate SD. Columns followed by the same letter are not significantly different (*P*<0.01). (C) Semi-quantitative RT-PCR gene expression analyses performed in roots of _His_AtRALF1-treated 10-d-old wild-type plants. Total RNA was extracted from roots of plants after 30min of treatment with different concentrations of the peptide. *GAPDH* expression was used as a control. *AtPRP1* and *AtPRP3*, proline-rich proteins 1 and 3 (*AtPRP1*, At1g54970 and *AtPRP3*, At3g62680); *AtHRGP2*, hydroxyproline-rich glycoprotein (At5g19800); *TCH4*, XET gene *TOUCH4* (At5g57560); *GAPDH*, glyceraldehyde-3-phosphate dehydrogenase (At1g13440). All experiments were repeated at least three times (independent biological replicates).

To understand the mechanism by which the AtRALF1 peptide inhibits cell elongation, we took advantage of an experiment comparing gene expression in 35S:AtRALF1 and wild-type seedlings whose results are deposited in the NCBI Gene Expression Omnibus (Edgar *et al.*, 2002) and are accessible using GEO Series accession number GSE641 (http://www.ncbi.nlm.nih.gov/geo/query/acc.cgi?acc=GSE641). We selected candidate genes to be validated using the following criteria: gene expression is altered in at least one sample; the gene is expressed in roots; and the gene is involved in cell elongation, growth, or expansion. We validated four genes, two that encode PRPs (*AtPRP1*, At1g54970, and *AtPRP3*, At3g62680), one that encodes a hydroxyproline-rich glycoprotein (here named *AtHRGP2*, At5g19800), and the XET gene *TOUCH4* (*TCH4*, At5g57560). All of these genes are upregulated in 35S:AtRALF1 plants, as predicted by the microarray data and confirmed by RT-PCR and qRT-PCR (Fig. 3A, B, see Supplementary Table S2, available at *JXB* online for ratios of the band intensities). Of the four AtRALF1-upregulated genes, *AtPRP1* and *TCH4* were also downregulated in irAtRALF1 plants. *AtPRP3* and *AtHRGP2* transcript levels in wild-type and irAtRALF1 plants were not significantly different. At least two reasons could be proposed to explain the normal expression levels of the *AtPRP3* and *AtHRGP2* genes in the irAtRALF1 plants. The first is that the residual level of *AtRALF1* expression in our knockdown line could be sufficient to support detectable levels of both *AtPRP3* and *AtHRGP2* expression. The second reason is related to the 37 RALF isoforms in the *Arabidopsis* genome and the possible functional redundancy of these genes. The lack of specificity for the *Arabidopsis* RALF isoforms can be inferred from the similarity of the independently obtained semi-dwarf phenotypes caused by the overexpression of AtRALF1, AtRALF23, or AtRALF8 (Matos *et al.*, 2008; Srivastava *et al.*, 2009; Atkinson *et al.*, 2013). In our irAtRALF1 line, we showed that the closest AtRALF isoforms were not affected by the AtRALF1 RNA interference construct (Supplementary Fig. S3 available at *JXB* online); in this case, any other AtRALF isoform that is expressed in roots could be responsible for maintaining the normal *AtPRP3* and *AtHRGP2* gene expression levels. Wild-type plants treated with exogenous _His_AtRALF1 exhibited the induction of all four genes in a _His_AtRALF1-concentration dependent manner (Fig. 3C, see Supplementary Table S2, available at *JXB* online, for ratios of the band intensities). Gene induction was rapid and could be detected by RT-PCR in as little as 30min using 0.1 μM _His_AtRALF1. After 3h of exposure to the peptide, all genes, except *TCH4*, returned to basal levels (Supplementary Fig. S4 available at *JXB* online). Both the fast response and the fact that expression returned to control levels after only 3h suggested a rapid turnover of the peptide or a mechanism of desensitization. The concentration dependence also suggested that the induction over time may be dependent on the availability of the signal. Desensitization has been shown for the peptide systemin (Yalamanchili and Stratmann, 2002), and the rapid degradation of RALF peptides in poplar cell culture medium has also been suggested (Haruta and Constabel, 2003).

Cell expansion is the result of the deposition of new cell-wall material and the rearrangement of the existing cell walls. PRPs are structural cell-wall proteins that have been linked to the wound response and to plant development (Tierney *et al.*, 1988; Hong *et al.*, 1990; Carpita and Gibeaut, 1993; Showalter, 1993). The *AtPRP1* and *AtPRP3* genes are expressed only in roots and are most likely involved in epidermal cell differentiation (Fowler *et al.*, 1999; Bernhardt and Tierney, 2000; Bruex *et al.*, 2012). Although its function is still unknown, the *AtHRGP2* gene was induced 2h after treatment with cytokinin (Lee *et al.*, 2007), and its transcript was detected at high levels in the *cl3egl3* mutant, which produces excessive root-hair cells. It was also found as a low-level transcript in *cpctry*, a non-hair-cell mutant line (Bruex *et al.*, 2012). The *TCH4* gene encodes a XET involved in cell-wall rearrangement and is expressed in young expanding leaves, lateral root primordial, and elongating hypocotyls, among other tissues (Xu *et al.*, 1995). The *TCH4* gene is regulated by several environmental stimuli such as cold, heat, and touch, as well as by hormones such as auxin and BRs. Auxin-stimulated induction occurs after 30min, similar to AtRALF1 induction, whereas BR induction occurs later and is detectable only 2h after treatment (Xu *et al.*, 1995 and Supplementary Fig. S5 available at *JXB* online). The identity of the AtRALF1-induced genes suggested that the effect of RALF in plant tissues may be a product of cell-wall rearrangement resulting in wall stiffening. High levels of the peptide in 35S:AtRALF1 plants and in plants treated with exogenous _His_AtRALF1 could cause premature wall hardening, which would prevent full elongation and result in smaller cells and plants.

### AtRALF1 opposes BL effects

Mingossi *et al.* (2010) showed that SacRALF peptides isolated from sugarcane are probably involved in cell expansion because their coding genes are expressed in expanding tissues. However, it is unclear why a peptide whose actions lead to a halt in cell elongation is located in regions of cell expansion. We hypothesize that RALF counteracts other signals that promote cell elongation. Among the signals that have been associated with cell elongation, BR is a good candidate because BL has been shown to downregulate *AtRALF23* gene expression, and plants transformed with the *AtRALF23* gene under the control of the 35S promoter exhibited impaired BL-induced hypocotyl elongation (Srivastava *et al.*, 2009). As opposed to *AtRALF23*, *AtRALF1* gene expression is not affected by BL (Srivastava *et al.*, 2009).

**Fig. 4.**
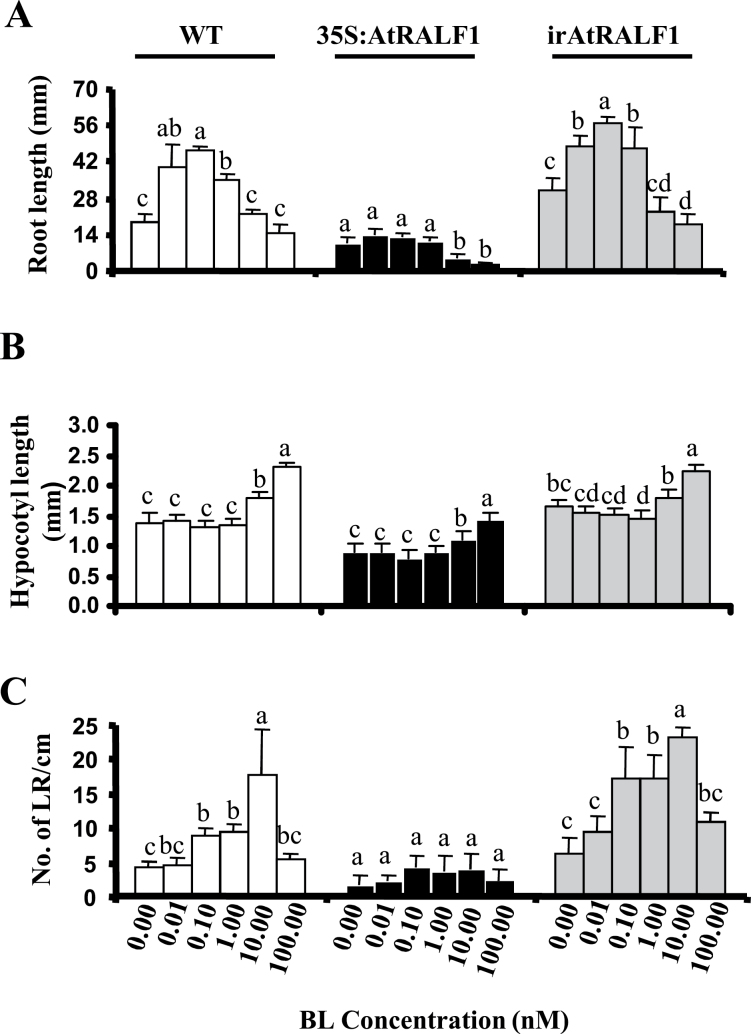
AtRALF1-overexpressing (35S:AtRALF1, black columns), AtRALF1-silencing (irAtRALF1, grey columns) transgenic lines and wild-type (WT) plants (white columns) treated with different concentrations of BL. (A) Root length. (B) Hypocotyl length of light-grown plants. (C) Number of emerged lateral roots per cm. Root length, hypocotyl length, and number of emerged lateral roots were measured in 10-d-old seedlings (*n*>30) grown in the presence of different concentrations of BL. Error bars indicate SD. Statistical analysis was made for each genetic background separately. Columns within each genotype that are followed by the same letter are not significantly different (*P*<0.01). All experiments were repeated at least three times.

Primary root elongation, hypocotyl elongation, and lateral root formation are three well-known effects of exogenously applied BR in *Arabidopsis* plants (Müssig *et al.*, 2003; Bao *et al.*, 2004; Clouse, 2011). BL induces root growth at 0.01, 0.1, and 1nM concentrations (Sasse, 1994; Müssig *et al.*, 2003). Transgenic plants overexpressing *AtRALF1* (35S:AtRALF1) were less sensitive to root growth-stimulating doses of BL, whereas plants with low levels of *AtRALF1* (irAtRALF1) were sensitive to the same concentrations (Fig. 4A). When compared with untreated plants, irAtRALF1 and wild-type plants showed 150 and 200% average increases in root growth, respectively, when treated with 0.1nM BL (Fig. 4A). When both 35S:AtRALF1 and irAtRALF1 transgenic plants were exposed to higher concentrations of BL (10 and 100nM), the characteristic inhibitory effect of BL on root growth was observed (Fig. 4A, and Roddick *et al.*, 1993; Müssig *et al.*, 2003). BRs act as inhibitors if a threshold level is exceeded, and this threshold level is dependent on the genotype (Müssig *et al.*, 2003); the 35S:AtRALF1 genotype was more sensitive to inhibitory concentrations of BL. Roots from 35S:AtRALF1 plants treated with 10nM BL were shorter than those from untreated plants (0 BL), whereas wild-type and irAtRALF1 plants exposed to the same concentration did not differ from the untreated plants, indicating that the threshold was lower in the 35S:AtRALF1 plants than in wild-type plants (Fig. 4A).

BL also induces hypocotyl elongation in light conditions (Mandava, 1988; Sasse, 1990; Clouse and Sasse, 1998). The BL-induced hypocotyl elongation response was 5 and 10% smaller in 35S:AtRALF1 plants exposed to 10 and 100nM BL, respectively, than in wild-type plants. However, the concentrations that caused elongation were the same within each genotype (Fig. 4B). Plants overexpressing *AtRALF1* already have shorter hypocotyls (64.3% of wild type), and when both 35S:AtRALF1 and wild-type plants were treated with BL, 35S:AtRALF1 plants had hypocotyls that were 56.5% smaller than those of wild-type plants (Fig. 4B). A similar response was reported for plants that overexpress *AtRALF23* (Srivastava *et al.*, 2009). Hypocotyls of light-grown irAtRALF1 plants are larger than hypocotyls of wild-type plants. In addition, when irAtRALF1 plants are exposed to BL, they elongate further but do not surpass the size of the hypocotyls of BL-treated wild-type plants, suggesting the lack of additive effects (Fig. 4B). One reason for the limited hypocotyl elongation response in irAtRALF1 plants could be that the cells had reached the limit of elongation in our experimental conditions, and even with an additional stimulus, they would not have been able to overcome light-mediated inhibition. A similar response has been described for auxin-overproducing plants (Romano *et al.*, 1995). Although the *AtRALF1* gene is mainly expressed in roots, we noticed that 35S:AtRALF1 plants, due to ectopic expression of the *AtRALF1* gene, also exhibited a compromised sensitivity to BL when rosette width and leaf length were evaluated (Supplementary Fig. S6 available at *JXB* online).

BL increases the lateral root number in *Arabidopsis* plants (Bao *et al.*, 2004). Plants overexpressing *AtRALF1* already have a reduced number of emerged lateral roots, and this number remained unchanged even upon treatment with high concentrations (e.g. 10nM) of BL (Fig. 4C). AtRALF1-silenced plants, which already have a large number of emerged lateral roots, exhibited further increases once treated with BL (Fig. 4C). Bao *et al.* (2004) showed that BL and auxin act synergistically to promote increased lateral root numbers. However, auxin appears to be a central player in the regulation of lateral root formation (Blakely *et al.*, 1982; Casimiro *et al.*, 2001; Péret *et al.*, 2009). To investigate if the peptide effect also opposes auxins in the formation of lateral roots, the transgenic plants were treated with IAA. Both overexpression (35S:AtRALF1) and suppression (irAtRALF1) of the peptide had no effect on IAA treatment, as both genotypes showed increased lateral root formation upon IAA treatment (Supplementary Fig. S7 available at *JXB* online). Our data regarding the effects of BL and IAA on lateral root formation showed that RALF opposes only BL and not IAA, suggesting that AtRALF1 acts downstream of or in parallel with IAA.

**Fig. 5.**
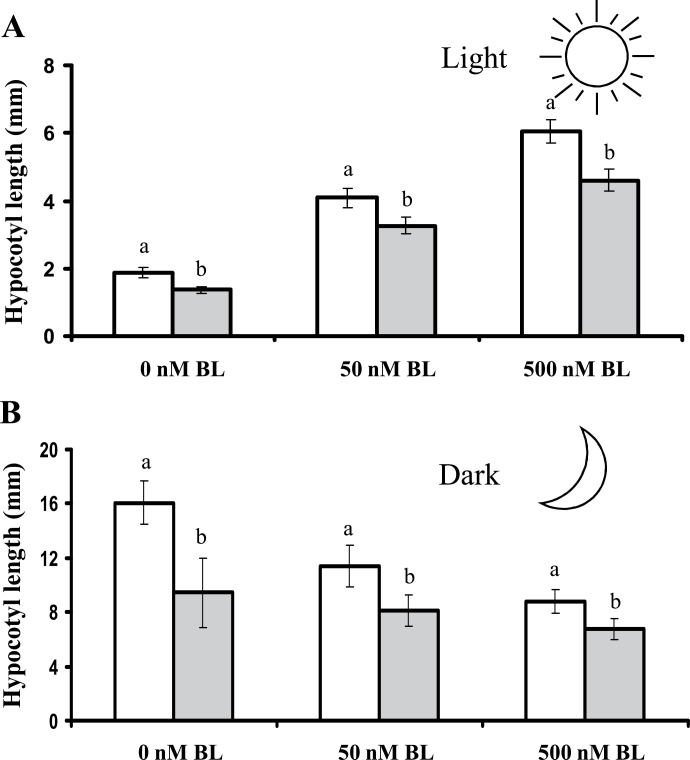
AtRALF1 effect on hypocotyl elongation in the presence or absence of BL at high concentrations. (A) Hypocotyl length of light-grown 5-d-old seedlings _His_AtRALF1 treated (1 µM, grey columns) or untreated (control, white columns). (B) Hypocotyl elongation of dark-grown 5-d-old seedlings _His_AtRALF1 treated (1 µM, grey columns) or untreated (control, white columns). Error bars indicate SD. Within each concentration of BL, columns followed by the same letter are not significantly different (*P*<0.01). All experiments were repeated at least three times.

BL has a different and opposite effect on hypocotyl elongation when it is applied under dark rather than light conditions. Specifically, BL promotes elongation when seedlings are exposed to light and inhibits elongation when seedlings are grown in the dark (Mandava, 1988; Turk *et al.*, 2003). To analyse whether the opposing effects of AtRALF1 and BL would be maintained even under conditions in which BL has an inhibitory effect, we simultaneously treated seedlings with _His_AtRALF1 and high concentrations of BL (50 and 500nM) under both light and dark conditions. _His_AtRALF1 decreased hypocotyl elongation in plants grown in both light and dark conditions (Fig. 5). In light conditions, _His_AtRALF1 slightly inhibited the full effect of BL-induced hypocotyl elongation, even at high BL concentrations such as 500nM (Fig. 5A). In etiolated plants, in which BL inhibits hypocotyl elongation (Turk *et al.*, 2003), treatment with the _His_AtRALF1 peptide and BL led to an even greater decrease in elongation, demonstrating the characteristics of an additive effect (Fig. 5B).

BL is known as a growth-promoting hormone, and a role for RALF in the regulation of cell expansion has also been proposed. Our data showed opposite roles for AtRALF1 and BL during primary root elongation, hypocotyl elongation, and lateral root formation. An additive effect of BL and AtRALF1 was only observed on the inhibition of hypocotyl elongation when high concentrations of BL were applied to dark-grown seedlings. Overall, these results are consistent with independent, opposite effects of BR and AtRALF1 on cell elongation.

### AtRALF1 induces genes of the BL biosynthetic pathway

**Fig. 6.**
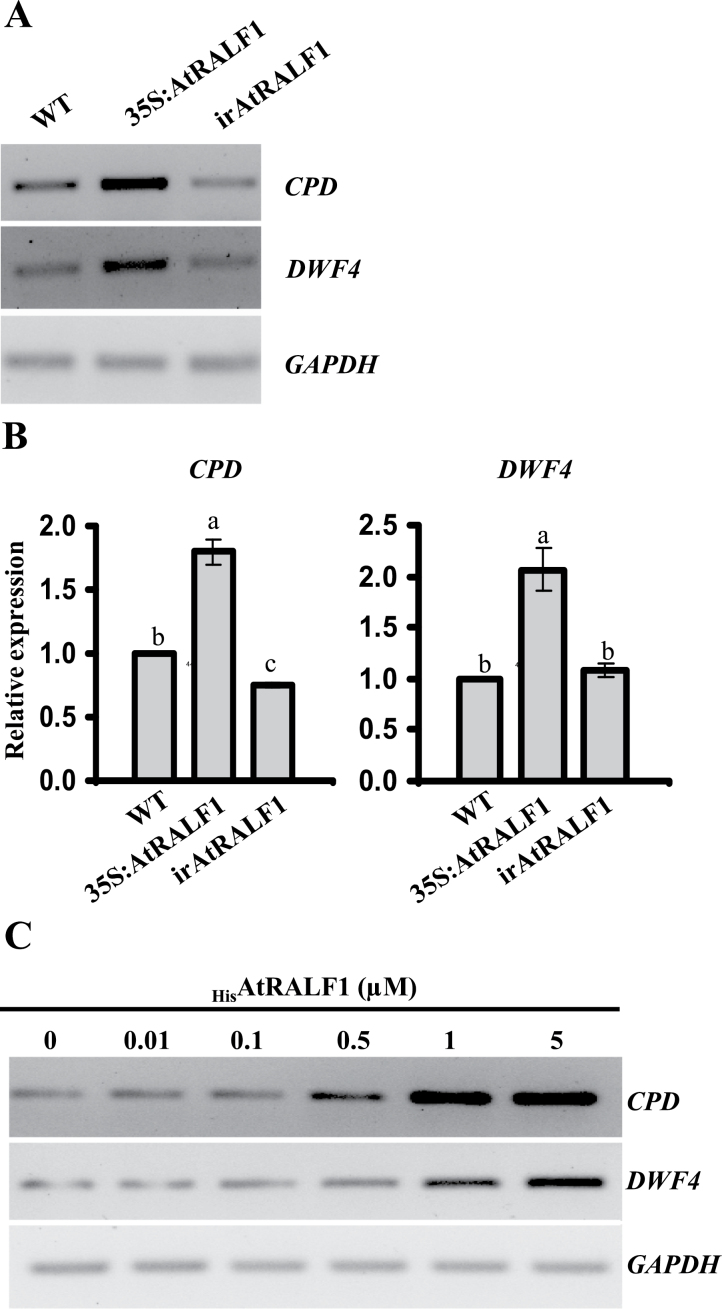
AtRALF1 effect on BL-downregulated genes *CPD* (At5g05690) and *DWF4* (At3g50660). (A, B) Semi-quantitative RT-PCR gene expression analyses performed in roots of 10-d-old AtRALF1-overexpressing (35S:AtRALF1) and AtRALF1-silenced (irAtRALF1) transgenic lines and wild-type plants (A) and quantitative qRT-PCR gene expression analyses (B). Error bars indicate SD. Columns followed by the same letter are not significantly different (*P*<0.01). (C) Semi-quantitative RT-PCR gene expression analyses performed in roots of _His_AtRALF1-treated 10-d-old wild-type plants. Total RNA was extracted from roots of plants after 30min of treatment with different concentrations of the peptide. *GAPDH* (At1g13440) expression was used as a control. All experiments were repeated at least three times (independent biological replicates).

To investigate further the effect of AtRALF1 on BL responses, we evaluated two BL-downregulated genes that encode cytochrome P450 monooxygenases, *CONSTITUTIVE PHOTOMORPHISM AND DWARFISM* (*CPD*) and *DWARF4* (*DWF4*), both of which are involved in the biosynthesis of BRs (Mathur *et al.*, 1998; Goda *et al.*, 2002). Roots from plants that overexpressed *AtRALF1* showed high levels of *CPD* and *DWF4* mRNA (Fig. 6A, B; see Supplementary Table S2 (available at *JXB* online) for ratios of the band intensities). In irAtRALF1 plants, the mRNA levels of the *CPD* gene were lower than in the wild type, whereas transcript levels for *DWF4* were similar to those of the wild type (Fig. 6A, B). When *Arabidopsis* plants were exposed to increasing concentrations of exogenously applied _His_AtRALF1, the *CPD* and *DWF4* genes also exhibited increased expression in roots, as demonstrated by RT-PCR (Fig. 6C; see Supplementary Table S2 (available at *JXB* online) for ratios of the band intensities). The gene expression analyses of two BL-downregulated genes again showed opposite roles for AtRALF1 and BL. The AtRALF1-mediated induction of these genes could be one way that plants have evolved to counteract excess RALF, perhaps by seeking a physiological equilibrium between RALF and BL. Coordination of the cellular progression mediated by BR in roots demands a refined balance of this hormone (González-García *et al.*, 2011; Hacham *et al.*, 2011). The compromised sensitivity to BL of the plants overexpressing AtRALF1 and the AtRALF1 induction of two genes involved in BR biosynthesis also suggest that AtRALF1 may be used as a signal to counteract BR-regulated cell growth.

### BL decreases the mRNA levels of genes upregulated by AtRALF1

**Fig. 7.**
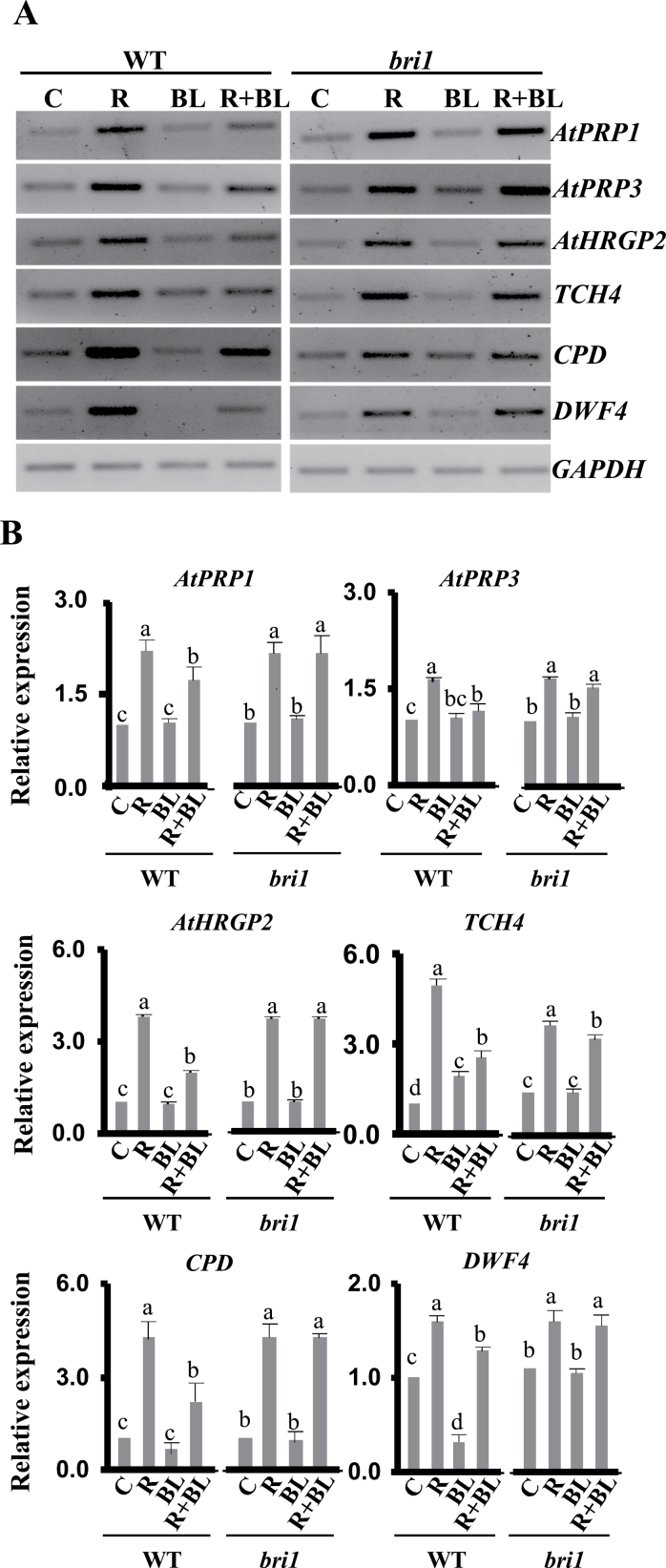
Gene expression analyses of AtRALF1-inducible genes upon simultaneous treatment with _His_AtRALF1 (R, 1 μM for 30min) and BL (1 μM for 30min). (A) Semi-quantitative RT-PCR performed using total RNA extracted from roots of untreated (C, control plants) or treated (R, BL, or R+BL) 10-d-old wild-type (WT) or BL-insensitive mutant *bri1* plants. (B) qRT-PCR performed using total RNA extracted from roots of untreated (C, control plants) or treated (R, BL, or R+BL) 10-d-old wild-type (WT) or BL-insensitive mutant *bri*1 plants. *GAPDH* expression was used as a control. *AtPRP1* and *AtPRP3*, proline-rich proteins 1 and 3 (*AtPRP1*, At1g54970, and *AtPRP3*, At3g62680); *AtHRGP2*, hydroxyproline-rich glycoprotein (At5g19800); *TCH4*, xyloglucan endotransglucosylase *TOUCH4* (At5g57560); *CPD*, constitutive photomorphism and dwarfism (At5g05690). *DWF4*, DWARF4 (At3g50660); *GAPDH*, glyceraldehyde-3-phosphate dehydrogenase (At1g13440). *DWF4* and *AtPRP1* were amplified using 26 and 29 PCR cycles, respectively. Error bars indicate SD. Statistical analysis was made for each genetic background separately. Columns within each genotype that are followed by the same letter are not significantly different (*P*<0.01). The experiment was performed at least three times (independent biological replicates).

Our study of plants simultaneously exposed to AtRALF1 and BL also suggested opposite effects of the two signals on cell elongation, and transgenic 35S:AtRALF1 plants showed a compromised response when treated with exogenous BL (Figs 4 and 5). To gain further insight into the molecular mechanism behind this inhibitory action of AtRALF1 on BL, we investigated how the expression of the AtRALF1-inducible genes, *AtPRP1*, *AtPRP3*, *AtHRGP2*, *TCH4*, *CPD*, and *DWF4*, would be affected upon simultaneous treatment with AtRALF1 and BL. BL treatment by itself had no effect on *AtPRP1*, *AtPRP3*, and *AtHRGP2* gene expression and, as expected, induced *TCH4* at later time points and downregulated *DWF4* (Fig. 7 and Supplementary Fig. S5, available at *JXB* online; see Supplementary Table S2, available at *JXB* online, for ratios of the band intensities) (Xu *et al.*, 1995; Mathur *et al.*, 1998; Goda *et al.*, 2002). The downregulation of *CPD* was not statistically significant at 30min but was clear at the 3h time point (Supplementary Fig. S8 available at *JXB* online). *DWF4* was also downregulated by BL treatment at the 3h time point (Supplementary Fig. S8 available at *JXB* online). When plants were simultaneously treated with _His_AtRALF1 and BL, the *AtPRP1*, *AtPRP3*, and *AtHRGP2* genes were induced but to a lesser degree compared with the induction by _His_AtRALF1 treatment alone (Fig. 7A, B). The *TCH4* gene was induced faster by _His_AtRALF1 than by BL treatment, and the simultaneous addition of _His_AtRALF1 and BL resulted in an intermediate level of induction (Fig. 7A, B). Xu *et al.* (1995) reported that *TCH4* mRNA levels were weakly induced 30min after BL treatment. *AtPRP1*, *AtPRP3*, *AtHRGP2*, *CPD*, and *DWF4* mRNAs all returned to control levels by 3h after _His_AtRALF1 treatment (Supplementary Fig. S8 available at *JXB* online). The *TCH4* gene remained induced until the end of the experiment (3h).

*TCH4* gene induction has been studied in a BR-insensitive mutant (*bri1-2*), and it has been reported that the signalling pathways that lead to the activation of this gene are independent or at least convergent at a point downstream of the BR receptor (Iliev *et al.*, 2002). This seems to be the case for the AtRALF1-mediated induction of the *TCH4* gene because its response is not compromised in the *bri1* mutant. AtRALF1 and BL may share a subset of transcription factors responsible for *TCH4* induction. The sharing of different regulatory elements was reported in a promoter deletion study of the *TCH4* gene (Iliev *et al.*, 2002). The sharing of transcription factors could also explain the lower level of induction of AtRALF1-inducible genes upon simultaneous treatment with RALF and BL. The recruitment of shared transcription factors by BL could limit a full AtRALF1 response.

When evaluated after simultaneous treatment with the peptide and BL, the BR biosynthetic genes *CPD* and *DWF4* also showed reduced mRNA expression levels when compared with the _His_AtRALF1 treatment alone (Fig. 7A, B). To confirm that BR sensing is required to minimize the induction of the RALF-inducible genes, we evaluated the combined responses in the *bri1* mutant (Clouse *et al.*, 1996). Although *bri1* mutant plants have proportionally shorter roots, treatment with the AtRALF1 peptide showed an even higher inhibition of root growth (Supplementary Fig. S9 available at *JXB* online). *bri1* plants exposed to the same treatments performed in the wild type showed that *bri1* only responded to _His_AtRALF1, and no reduction in gene expression was observed upon simultaneous BL/_His_AtRALF1 treatment (Fig. 7A, B). The *TCH4* gene was the only one to show a minor decrease in *bri1* plants treated with both _His_AtRALF1 and BL. *TCH4* responds to a complex set of signals, including auxin, BRs, environmental stimuli, and development. The inhibition of a full AtRALF1 response when *bri1* plants are treated with _His_AtRALF1 and BL could mean that BL is perhaps inhibiting AtRALF1 through one of the other BL receptors that have specific functions in cell growth (Caño-Delgado *et al.*, 2004).

### Supplementary data

Supplementary data are available at *JXB* online.

Supplementary Fig. S1. Exogenous _His_AtRALF1 effect on root length and hypocotyl elongation in irAtRALF1 plants.

Supplementary Fig. S2. *AtRALF1*-overexpressing (35S:AtRALF1) and *AtRALF1*-silenced (irAtRALF1) transgenic lines.

Supplementary Fig. S3. Semi-quantitative RT-PCR gene expression analyses performed in roots of 10-d-old AtRALF1-silenced (irAtRALF1) and wild-type (WT) plants.

Supplementary Fig. S4. Semi-quantitative RT-PCR gene expression analyses performed in roots of untreated (WT) and _His_AtRALF1-treated 10-d-old wild-type plants.

Supplementary Fig. S5. Time course analysis of the xyloglucan endotransglucosylase *TOUCH4* (At5g57560) gene expression after treatment with auxin, _His_AtRALF1 and brassinolide.

Supplementary Fig. S6. *AtRALF1*-overexpressing (35S:AtRALF1, black columns) and *AtRALF1*-silenced (irAtRALF1, grey columns) transgenic lines treated with different concentrations of brassinolide (BL).

Supplementary Fig. S7. Number of emerged lateral roots in *AtRALF1*-overexpressing (35S:AtRALF1, black columns) and *AtRALF1*-silenced (irAtRALF1, grey columns) transgenic lines treated with brassinolide (BL) or indole-3-acetic acid (IAA).

Supplementary Fig. S8. Gene expression analyses of AtRALF1-inducible genes upon simultaneous treatment with _His_AtRALF1 (R, 1 μM for 30min) and brassinolide (BL, 1 μM for 30min).

Supplementary Fig. S9. _His_AtRALF1 effect on root length in *bri1* mutants.

Supplementary Table S1. Primers used for cloning, quantitative and semi-quantitative RT-PCR analyses.

Supplementary Table S2. Evaluation of RT-PCR signals via densitometry.

## Supplementary Material

Supplementary Data

## Abbreviations:

BL: brassinolide
BR: brassinosteroid
CT: threshold cycle
GAPDH: glyceraldehyde 3-phosphate dehydrogenase
HRGP: hydroxyproline-rich glycoprotein
IAA: indole-3-acetic acid
MS: Murashige and Skoog
PRP: proline-rich protein
RALF: rapid alkalinization factor
SD: standard deviation.

## References

[CIT0001] Atkinson NJ Lilley CJ Urwin PE 2013 Identification of genes involved in the response of Arabidopsis to simultaneous biotic and abiotic Stresses. Plant Physiology 162, 2028–2041 23800991 10.1104/pp.113.222372 PMC3729780

[CIT0002] Bao F Shen J Brady SR Muday GK Asami T Yang Z 2004 Brassinosteroids interact with auxin to promote lateral root development in *Arabidopsis* . Plant Physiology 134, 1624–1631 15047895 10.1104/pp.103.036897 PMC419836

[CIT0003] Benatti MR Penning BW Carpita NC McCann MC 2012 We are good to grow: dynamic integration of cell wall architecture with the machinery of growth. Frontiers in Plant Science 3, 1–6 22936938 10.3389/fpls.2012.00187 PMC3424494

[CIT0004] Bernhardt C Tierney M 2000 Expression of AtPRP3, a proline-rich structural cell wall protein from *Arabidopsis*, is regulated by cell-type-specific developmental pathways involved in root hair formation. Plant Physiology 122, 705–714 10712533 10.1104/pp.122.3.705 PMC58905

[CIT0005] Birnbaum K Shasha DE Wang JY Jung JW Lambert GM Galbraith DW Benfey PN 2003 A gene expression map of the Arabidopsis root. Science 302, 1956–1960 14671301 10.1126/science.1090022

[CIT0006] Blakely LM Durham M Evans TA Blakely RM 1982 Experimental studies on lateral root formation in radish seedling roots: I. General methods, developmental stages, and spontaneous formation of laterals. Botanical Gazette 143, 341–352

[CIT0007] Boller T 2005 Peptide signaling in plant development and self/non-self perception. Current Opinion in Cell Biology 17, 116–122 15780586 10.1016/j.ceb.2005.02.007

[CIT0008] Bruex A Kainkaryam RM Wieckowski Y 2012 A gene regulatory network for root epidermis cell differentiation in *Arabidopsis* . PLoS Genetics 8, 1–20 10.1371/journal.pgen.1002446 PMC3257299 22253603

[CIT0009] Caño-Delgado A Yin Y Yu C Vafeados D Mora-García S Cheng JC Nam KH Li J Chory J 2004 BRL1 and BRL3 are novel brassinosteroid receptors that function in vascular differentiation in *Arabidopsis* . Development 131, 5341–5351 15486337 10.1242/dev.01403

[CIT0010] Carpita NC Gibeaut DM 1993 Structural models of primary cell walls in flowering plants: consistency of molecular structure with the physical properties of the walls during growth. The Plant Journal 3, 1–30 8401598 10.1111/j.1365-313x.1993.tb00007.x

[CIT0011] Casimiro I Marchant A Bhalerao RP 2001 Auxin transport promotes *Arabidopsis* lateral root initiation. The Plant Cell 13, 843–852 11283340 10.1105/tpc.13.4.843 PMC135543

[CIT0012] Chinchilla D Shan L He P de Vries S Kemmerling B 2009 One for all: the receptor-associated kinase BAK1. Trends in Plant Science 14, 535–541 19748302 10.1016/j.tplants.2009.08.002 PMC4391746

[CIT0013] Clough SJ Bent AS 1998 Floral dip: a simplified method for Agrobacterium-mediated transformation of *Arabidopsis thaliana* . The Plant Journal 16, 735–743 10069079 10.1046/j.1365-313x.1998.00343.x

[CIT0014] Clouse SD 2004 Brassinosteroid signal transduction and action. In: Davies PJ, ed. Plant hormones, biosynthesis, signal transduction, action. Dordrecht, The Netherlands: Kluwer Academic Publishers, 204–220

[CIT0015] Clouse SD 2011 Brassinosteroid signal transduction: from receptor kinase activation to transcriptional networks regulating plant development. Plant Cell 23, 1219–1230 21505068 10.1105/tpc.111.084475 PMC3101532

[CIT0016] Clouse SD Langford M McMorris TC 1996 A brassinosteroid-insensitive mutant in *Arabidopsis thaliana* exhibits multiple defects in growth and development. Plant Physiology 111, 671–678 8754677 10.1104/pp.111.3.671 PMC157882

[CIT0017] Clouse SD Sasse JM 1998 Brassinosteroids: essential regulators of plant growth and development. Annual Reviews of Plant Physiology and Plant Molecular Biology 49, 427–451 10.1146/annurev.arplant.49.1.427 15012241

[CIT0018] Cosgrove DJ 1999 Enzymes and other agents that enhance cell wall extensibility. Annual Reviews of Plant Physiology and Plant Molecular Biology 50, 391–417 10.1146/annurev.arplant.50.1.391 11541953

[CIT0019] Cosgrove DJ 2005 Growth of the plant cell wall. Nature Reviews Molecular Cell Biology 6, 850–861 10.1038/nrm1746 16261190

[CIT0020] Covey PA Subbaiah CC Parsons RL Pearce G Lay FT Anderson MA Ryan CA Bedinger PA 2010 A pollen-specific RALF from tomato that regulates pollen tube elongation. Plant Physiology 153, 703–715 20388667 10.1104/pp.110.155457 PMC2879774

[CIT0021] Edgar R Domrachev M Lash AE 2002 Gene expression Omnibus: NCBI gene expression and hybridization array data repository. Nucleic Acids Research 30, 207–210 11752295 10.1093/nar/30.1.207 PMC99122

[CIT0022] Eklöf JM Brumer H 2012 The *XTH* gene family: an update on enzyme structure, function, and phylogeny in xyloglucan remodeling. Plant Physiology 153, 456–466 20421457 10.1104/pp.110.156844 PMC2879796

[CIT0023] Escobar NM Haupt S Thow G Boevink P Chapman S Oparka K 2003 High-throughput viral expression of cDNA–green fluorescent protein fusions reveal novel subcellular addresses and identifies unique proteins that interact with plasmodesmata. Plant Cell 15, 1507–1523 12837943 10.1105/tpc.013284 PMC165397

[CIT0024] Fowler TJ Bernhardt C Tierney M 1999 Characterization and expression of four proline-rich cell wall protein genes in *Arabidopsis* encoding two distinct subsets of multiple domain proteins. Plant Physiology 121, 1081–1091 10594096 10.1104/pp.121.4.1081 PMC59476

[CIT0025] Fry SC 2004 Primary cell wall metabolism: tracking the careers of wall polymers in living plant cells. New Phytologist 161, 641–645 10.1111/j.1469-8137.2004.00980.x 33873719

[CIT0026] Fry SC Smith RC Renwick KF Martin DJ Hodge SK Matthews KJ 1992 Xyloglucan endotransglycosylase, a new wall-loosening enzyme activity from plants. Biochemical Journal 282, 821–828 1554366 10.1042/bj2820821 PMC1130861

[CIT0027] Fu X Harberd NP 2003 Auxin promotes *Arabidopsis* root growth by modulating gibberellin response. Nature 421, 740–743 12610625 10.1038/nature01387

[CIT0028] Gendreau E Traas J Desnos T Grandjean O Caboche M Höfte H 1997 Cellular basis of hypocotyl growth in *Arabidopsis thaliana* . Plant Physiology 114, 195–205 10.1104/pp.114.1.295 PMC158305 9159952

[CIT0029] Goda H Shimada Y Asami T Fujioka S Yoshida S 2002 Microarray analysis of brassinosteroid-regulated genes in *Arabidopsis* . Plant Physiology 130, 1319–1334 12427998 10.1104/pp.011254 PMC166652

[CIT0030] González-García MP Vilarrasa-Blasi J Zhiponova M Divol F Mora-García S Russinova E Caño-Delgado AI 2011 Brassinosteroids control meristem size by promoting cell cycle progression in Arabidopsis roots. Development 138, 849–859 21270057 10.1242/dev.057331

[CIT0031] Hacham Y Holland N Butterfield C Ubeda-Tomas S Bennett MJ Chory J Savaldi-Goldstein S 2011 Brassinosteroid perception in the epidermis controls root meristem size. Development 138, 839–848 21270053 10.1242/dev.061804 PMC3035089

[CIT0032] Haruta M Constabel CP 2003 Rapid alkalinization factors in poplar cell cultures. Peptide isolation, cDNA cloning, and differential expression in leaves and methyl jasmonate-treated cells. Plant Physiology 131, 814–823 12586905 10.1104/pp.014597 PMC166857

[CIT0033] Haruta M Monshausen G Gilroy S Sussman MR 2008 A cytoplasmic Ca^2+^ functional assay for identifying and purifying endogenous cell signaling peptides in *Arabidopsis* seedlings: identification of AtRALF1 peptide. Biochemistry 47, 6311–6321 18494498 10.1021/bi8001488

[CIT0034] He Z Wang Z Li J Zhu Q Lamb C Ronald P Chory J 2000 Perception of brassinosteroids by the extracellular domain of the receptor kinase BRI1. Science 288, 2360–2363 10875920 10.1126/science.288.5475.2360

[CIT0035] Hong JC Nagao RT Key JL 1990 Characterization of a proline-rich cell wall protein gene family of soybean. Journal of Biological Chemistry 265, 2470–2475 2303411

[CIT0036] Iliev EA Xu W Polisensky DH Oh M Torisky RS Clouse SD Braam J 2002 Transcriptional and posttranscriptional regulation of *Arabidopsis TCH4* expression by diverse stimuli. Roles of cis regions and brassinosteroids. Plant Physiology 130, 770–783 12376643 10.1104/pp.008680 PMC166605

[CIT0037] Karimi M Inzé D Depicker A 2002 GATEWAY vectors for Agrobacterium-mediated plant transformation. Trends in Plant Science 7, 193–195 11992820 10.1016/s1360-1385(02)02251-3

[CIT0038] Kieliszewski MJ Lamport DTA 1994 Extensin: repetitive motifs, functional sites, post-translational codes, and phylogeny. The Plant Journal 5, 157–172 8148875 10.1046/j.1365-313x.1994.05020157.x

[CIT0039] Lamesch P Berardini TZ Swarbreck D 2011 The *Arabidopsis* Information Resource (TAIR): improved gene annotation and new tools. Nucleic Acids Research 40, 1202–1210 10.1093/nar/gkr1090 PMC3245047 22140109

[CIT0040] Laplaze L Benkova E Casimiro I 2007 Cytokinins act directly on lateral root founder cells to inhibit root initiation. Plant Cell 19, 3889–3900 18065686 10.1105/tpc.107.055863 PMC2217640

[CIT0041] Lee DJ Park J Ku S Ha Y Kim S Kim MD Oh M Kim J 2007 Genome-wide expression profiling of *ARABIDOPSIS RESPONSE REGULATOR7* (*ARR7*) overexpression in cytokinin response. Molecular Genetics and Genomics 277, 115–137 17061125 10.1007/s00438-006-0177-x

[CIT0042] Lewis DR Negi S Sukumar P Muday GK 2011 Ethylene inhibits lateral root development, increases IAA transport and expression of PIN3 and PIN7 auxin efflux carriers. Development 138, 3485–3495 21771812 10.1242/dev.065102

[CIT0043] Li JM Jin H 2007 Regulation of brassinosteroid signaling. Trends in Plant Science 12, 37–41 17142084 10.1016/j.tplants.2006.11.002

[CIT0044] Livak K Schmittgen T 2001 Analyses of relative gene expression data using real-time quantitative PCR and the 2^–∆∆CT^ method. Methods 25, 402–408 11846609 10.1006/meth.2001.1262

[CIT0045] Malamy JE Benfey PN 1997 Organization and cell differentiation in lateral roots of *Arabidopsis thaliana* . Development 124, 33–44 9006065 10.1242/dev.124.1.33

[CIT0046] Mandava NB 1988 Plant Growth-promoting brassinosteroids. Annual Reviews of Plant Physiology and Plant Molecular Biology 39, 23–52

[CIT0047] Mathur J Molnár G Fujioka S 1998 Transcription of the *Arabidopsis CPD* gene, encoding a steroidogenic cytochrome P450, is negatively controlled by brassinosteroids. The Plant Journal 14, 593–602 9675902 10.1046/j.1365-313x.1998.00158.x

[CIT0048] Matos JM Fiori CS Silva-Filho MC Moura DS 2008 A conserved dibasic site is essential for correct processing of the peptide hormone AtRALF1 in *Arabidopsis thaliana* . FEBS Letters 582, 3343–3347 18775699 10.1016/j.febslet.2008.08.025

[CIT0049] Matsubayashi Y Sakagami Y 2006 Peptide hormones in plants. Annual Review of Plant Biology 57, 649–674 10.1146/annurev.arplant.56.032604.144204 16669777

[CIT0050] Mayumi K Shibaoka H 1995 A possible double role for brassinolide in the reorientation of cortical microtubules in the epidermal cells of Azuki bean epicotyls. Plant Cell Physiology 36, 173–181

[CIT0051] McCann MC Bush M Milione D 2001 Approaches to understanding the functional architecture of the plant cell wall. Phytochemistry 57, 811–821 11423133 10.1016/s0031-9422(01)00144-3

[CIT0052] Mingossi FB Matos JL Rizzato AP Medeiros AH Falco MC Silva-Filho MC Moura DS 2010 SacRALF1, a peptide signal from the grass sugarcane (*Saccharum* spp.), is potentially involved in the regulation of tissue expansion. Plant Molecular Biology 73, 271–281 20148351 10.1007/s11103-010-9613-8

[CIT0053] Moura DS Silva-Filho MC 2006 Plant peptide hormones, from defense to pollen self-incompatibility, cell fate and development: small peptides as signaling molecules in plants. In: Silva JAT, ed. Floriculture, ornamental and plant biotechnology: advances and topical issues. London: Global Science Books, 203–209

[CIT0054] Müssig C Shin GH Altmann T 2003 Brassinosteroids promote root growth in *Arabidopsis* . Plant Physiology 133, 1261–1271 14526105 10.1104/pp.103.028662 PMC281621

[CIT0055] Negi S Ivanchenko MG Muday GK 2008 Ethylene regulates lateral root formation and auxin transport in *Arabidopsis thaliana* . The Plant Journal 55, 175–187 18363780 10.1111/j.1365-313X.2008.03495.x PMC2635504

[CIT0056] Parry G Estelle M 2006 Auxin receptors: a new role for F-box proteins. Current Opinion in Cell Biology 18, 152–156 16488128 10.1016/j.ceb.2006.02.001

[CIT0057] Pearce G Moura DS Stratmann J Ryan CA 2001 RALF, a 5-kDa ubiquitous polypeptide in plants, arrests root growth and development. Proceedings of the National Academy of Sciences, USA 98, 12843–12847 10.1073/pnas.201416998 PMC60141 11675511

[CIT0058] Pearce G Yamaguchi Y Munske G Ryan CA 2010 Structure–activity studies of RALF, Rapid Alkalinization Factor, reveal an essential – YISY – motif. Peptides 31, 1973–1977 20800638 10.1016/j.peptides.2010.08.012

[CIT0059] Péret B Rybel BD Casimiro I Benková E Swarup R Laplaze L Beeckman T Bennett MJ 2009 *Arabidopsis* lateral root development: an emerging story. Trends in Plant Science 14, 399–408 19559642 10.1016/j.tplants.2009.05.002

[CIT0060] Roddick JG Rijnenberg AL Ikekawa N 1993 Developmental effects of 24-epibrassinolide in excised roots of tomato grown in vitro. Physiologia Plantarum 87, 453–458

[CIT0061] Romano CP Robson PRH Smith H Estelle M Klee H 1995 Transgene-mediated auxin overproduction in *Arabidopsis*: hypocotyl elongation phenotype and interactions with the *by6-1* hypocotyl elongation and *axrl* auxin-resistant mutants. Plant Molecular Biology 27, 1071–1083 7766890 10.1007/BF00020881

[CIT0062] Ryan CA Pearce G Scheer JM Moura DS 2002 Polypeptide hormones. Plant Cell 14, S251–S264 12045281 10.1105/tpc.010484 PMC151259

[CIT0063] Sasse JM 1990 Brassinolide-induced elongation and auxin. Physiologia Plantarum 80, 401–408

[CIT0064] Sasse JM 1994 Brassinosteroids and roots. Proceedings of the Plant Growth Regulation Society of America 21, 228–232

[CIT0065] Scheer JM Pearce G Ryan CA 2005 LeRALF, a plant peptide that regulates root growth and development, specifically binds to 25 and 120kDa cell surface membrane proteins of *Lycopersicon peruvianum* . Planta 221, 667–674 15909150 10.1007/s00425-004-1442-z

[CIT0066] Showalter AM 1993 Structure and function of plant cell wall proteins. Plant Cell 5, 9–23 8439747 10.1105/tpc.5.1.9 PMC160246

[CIT0067] Smalle J Haegman M Kurepa J Van Montagu M Straeten DV 1997 Ethylene can stimulate Arabidopsis hypocotyl elongation in the light. Proceedings of the National Academy of Sciences, USA 94, 2756–2761 10.1073/pnas.94.6.2756 PMC20163 11038610

[CIT0068] Srivastava R Liu JX Guo H Yin Y Howell SH 2009 Regulation and processing of a plant peptide hormone, AtRALF23, in *Arabidopsis* . The Plant Journal 59, 930–939 19473327 10.1111/j.1365-313X.2009.03926.x

[CIT0069] Steel RG Torrie JH Dickey DA 1996 Principles and procedures of statistics: a biometrical approach, 3rd edn New York: McGraw-Hill Companies

[CIT0070] Tierney ML Wiechart J Pluymers D 1988 Analysis of expression of expression and p33-related cell wall proteins in carrot and soybean. Molecular & General Genetics 211, 393–399

[CIT0071] Turk EM Fujioka S Seto H Shimada Y Takatsuto S Yoshida S Denzel MA Torres QI Neff MM 2003 CYP72B1 inactivates brassinosteroid hormones: an intersection between photomorphogenesis and plant steroid signal transduction. Plant Physiology 133, 1643–1653 14605216 10.1104/pp.103.030882 PMC300720

[CIT0072] Vanstraelen M Benková E 2012 Hormonal interactions in the regulation of plant development. Annual Review of Cell and Developmental Biology 28, 22.1–22.25 10.1146/annurev-cellbio-101011-155741 22856461

[CIT0073] Vert G Nemhauser JL Geldner N Hong F Chory J 2005 Molecular mechanisms of steroid hormone signaling in plants. Annual Review of Cell and Developmental Biology 21, 177–201 10.1146/annurev.cellbio.21.090704.151241 16212492

[CIT0074] Wang X Zhang J Yuan M Ehrhardt DW Wang Z Mao T 2012 *Arabidopsis* MICROTUBULE DESTABILIZING PROTEIN40 is involved in brassinosteroid regulation of hypocotyl elongation. Plant Cell 24, 4012–4025 23115248 10.1105/tpc.112.103838 PMC3517233

[CIT0075] Wang Z Nakano T Gendron J 2002 Nuclear-localized BZR1 mediates brassinosteroid-induced growth and feedback suppression of brassinosteroid biosynthesis. Developmental Cell 2, 505–513 11970900 10.1016/s1534-5807(02)00153-3

[CIT0076] Weigel D Glazebrook J 2002 Arabidopsis: a Laboratory Manual, 1st edn New York: Cold Spring Harbor Laboratory

[CIT0077] Winter D Vinegar B Nahal H Ammar R Wilson GV Provart NJ 2007 An “Electronic Fluorescent Pictograph” browser for exploring and analyzing large-scale biological data sets. PLoS One 8, e718 17684564 10.1371/journal.pone.0000718 PMC1934936

[CIT0078] Wu J Kurten EL Monshausen G Hummel GM Gilroy S Baldwin IT 2007 NaRALF, a peptide signal essential for the regulation of root hair tip apoplastic pH in *Nicotiana attenuata*, is required for root hair development and plant growth in native soils. The Plant Journal 52, 877–890 17916115 10.1111/j.1365-313X.2007.03289.x

[CIT0079] Xu W Purugganan MM Polisensky DH Antosiewicz DM Fry SC Braam J 1995 *Arabidopsis TCH4*, regulated by hormones and the environment, encodes a xyloglucan endotransglycosylase. Plant Cell 7, 1555–1567 7580251 10.1105/tpc.7.10.1555 PMC161010

[CIT0080] Yalamanchili RD Stratmann JW 2002 Ultraviolet-B activates components of the systemin signaling pathway in *Lycopersicon peruvianum* suspension-cultured cells. Journal of Biological Chemistry 32, 28424–28430 12034744 10.1074/jbc.M203844200

