# Supplementary material for: Arabidopsis thaliana RALF1 opposes brassinosteroid effects on root cell elongation and lateral root formation
Source: J Exp Bot. 2014 Mar 11;65(8):2219–30. doi: 10.1093/jxb/eru099 (PMC3991750; doi:10.1093/jxb/eru099)
Supplement: Supplementary Data [file supp_65_8_2219__index.html]

 Arabidopsis thaliana RALF1 opposes brassinosteroid effects on root cell elongation and lateral root formation — Arabidopsis thaliana RALF1 opposes brassinosteroid effects on root cell elongation and lateral root formation — Supplementary Data 

# *Arabidopsis thaliana* RALF1 opposes brassinosteroid effects on root cell elongation and lateral root formation

## Supplementary Data

Data files

**Files in this Data Supplement:**

- Supplementary Data - Supplementary Data
