# Supplementary material for: Arabidopsis thaliana RALF1 opposes brassinosteroid effects on root cell elongation and lateral root formation
Source: J Exp Bot. 2014 Mar 11;65(8):2219–30. doi: 10.1093/jxb/eru099 (PMC3991750; doi:10.1093/jxb/eru099)
Supplement: Supplementary Data [file supp_eru099_jexbot116343_file001.pdf]

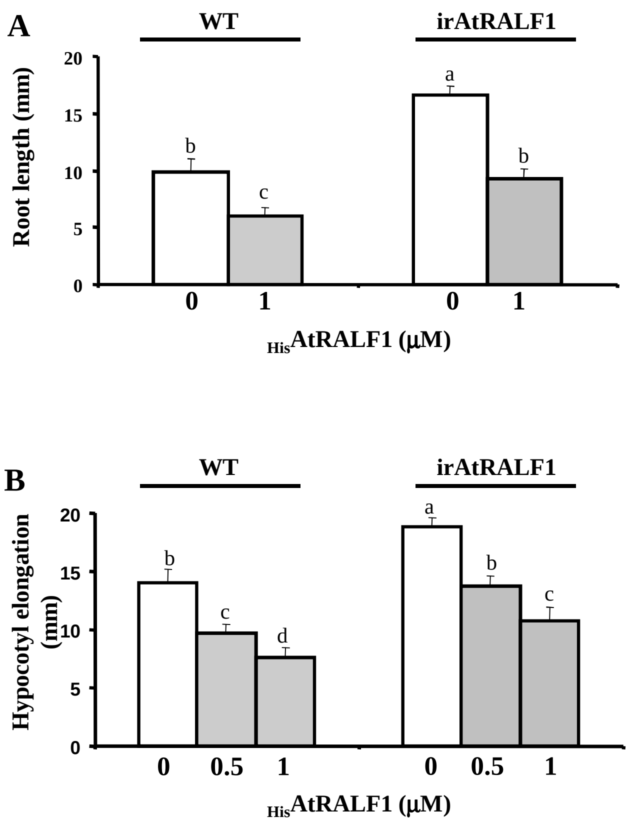

Supplementary Fig. S1. Exogenous $_{His}$AtRALF1 effect on root length and hypocotyl elongation in irAtRALF1 plants. (A) Root length of light-grown 5-d-old seedlings $_{His}$AtRALF1-treated (gray columns) or untreated (control, white columns). (B) Hypocotyl elongation of dark-grown 5-d-old seedlings $_{His}$AtRALF1-treated (gray columns) or untreated (control, white columns). Error bars indicate SD. Columns followed by the same letter are not significantly different (*p-value* < 0.01). The experiment was performed at least three times.

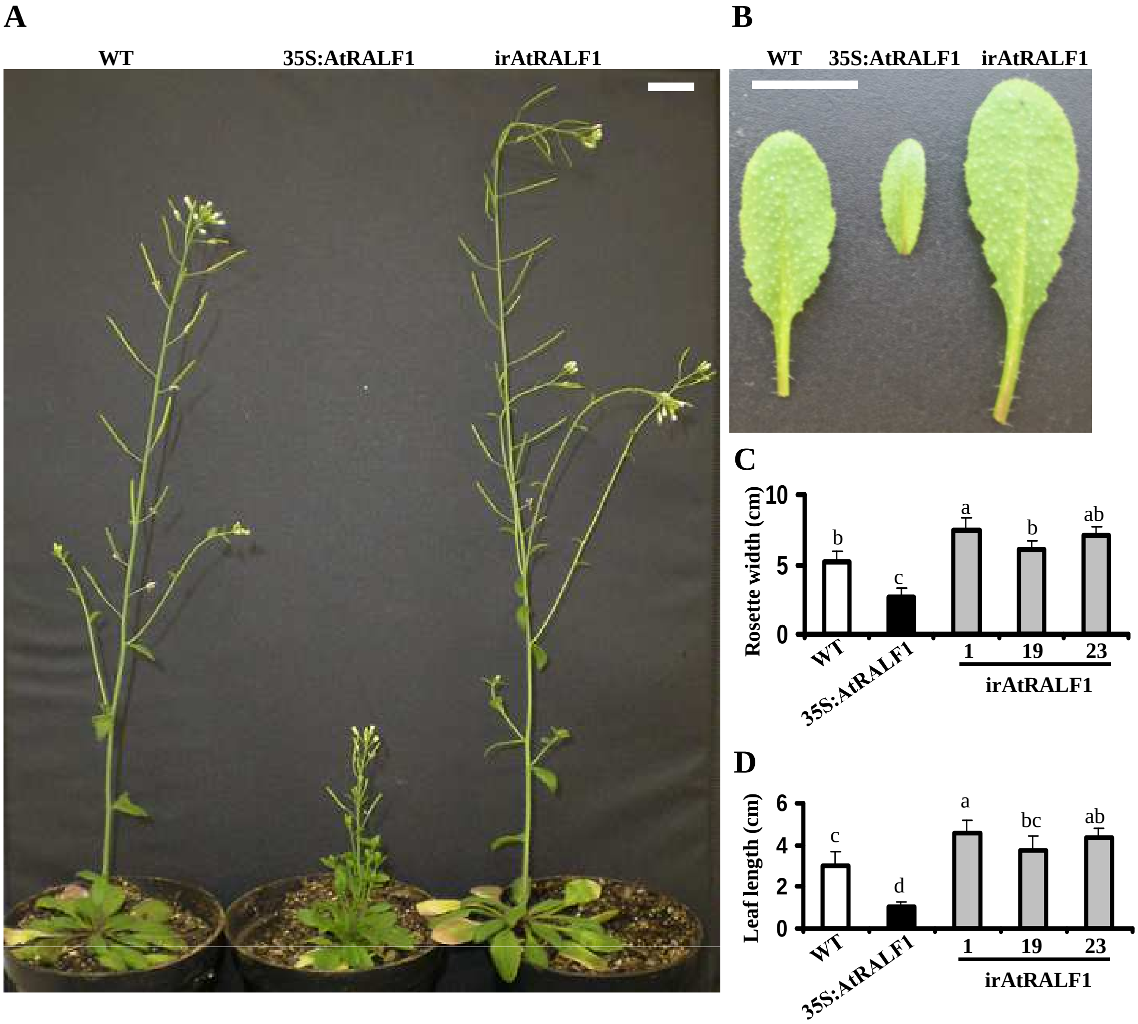

Supplementary Fig. S2. AtRALF1-overexpressing (35S:AtRALF1) and AtRALF1-silencing (irAtRALF1) transgenic lines. (A) Phenotype of 40-d-old plants. (B) Representative 7th leaf from wild type (left), 35S:AtRALF1 (center) and irAtRALF1 plants (right). (C) Rosette width of 40-d-old plants. (D) Length of the 7th leaf of 40-d-old plants. Bars = 1.5 cm. Error bars indicate SD. Columns followed by the same letter are not significantly different (*p-value* <0.01). The experiment was performed at least three times.

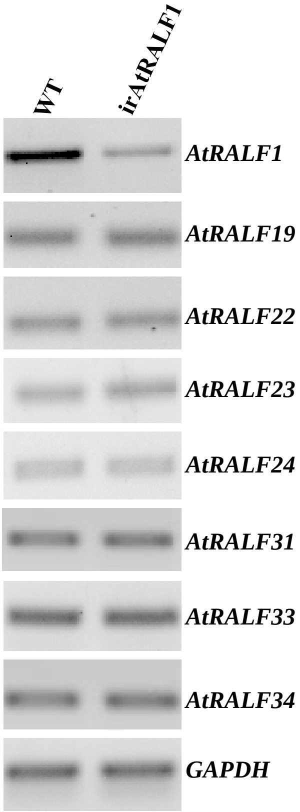

Supplementary Fig. S3. Semi-quantitative RT-PCR gene expression analyses performed in roots of 10-d-old AtRALF1-silecing (irAtRALF1) and wild type (WT) plants. *GAPDH* expression was used as a control. *AtRALF1* mRNA levels are shown in irAtRALF1 and WT for comparison. *AtRALF1*, *AtRALF19*, *AtRALF33*, *AtRALF34* and *GAPDH* amplification bands are shown after 27 PCR cycles. *AtRALF22*, *AtRALF23*, *AtRALF24* and *AtRALF31* amplification bands are shown after 31 PCR cycles. *AtRALF1* (At1g02900). *AtRALF19* (At2g33775). *AtRALF22* (At3g05490). *AtRALF23* (At3g16570). *AtRALF24* (At3g23805). *AtRALF31* (At4g13950). *AtRALF33* (At4g15800). *AtRALF34* (At5g67070). *GAPDH*, glycelraldehyde-3-phosphate dehydrogenase (At1g13440). The experiment was performed at least three times (independent biological replicates). See Supplementary Table S2 for ratios of the band intensities.

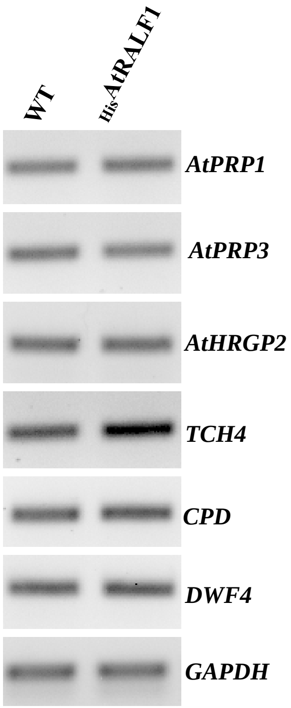

Supplementary Fig. S4. Semi-quantitative RT-PCR gene expression analyses performed in roots of untreated (WT) and $_{\text{His}}$AtRALF1-treated 10-d-old wild type plants. Total RNA was extracted from roots of wild type plants after 3 h treatment with 1 μM of $_{\text{His}}$AtRALF1. *GAPDH* expression was used as a control. *AtPRP1* and *AtPRP3*, proline-rich proteins 1 and 3 (*AtPRP1*, At1g54970 and *AtPRP3*, At3g62680). *AtHRGP2*, hydroxyproline-rich glycoprotein (At5g19800). *TCH4*, xyloglucan endotransglucosylase TOUCH4 (At5g57560). *CPD*, constitutive photomorphism and dwarfism (At5g05690). *DWF4*, DWARF4 (At3g50660). *GAPDH*, glyceraldehyde-3- phosphate dehydrogenase (At1g13440). The experiment was performed at least three times (independent biological replicates). See Supplementary Table S2 for ratios of the band intensities.

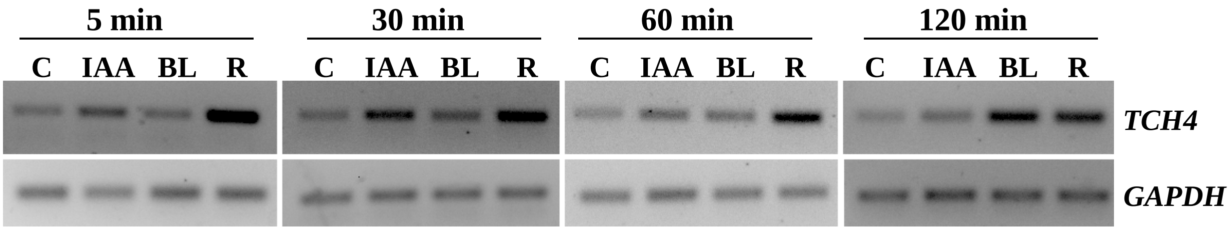

Supplementary Fig. S5. Time course analysis of the xyloglucan endotransglucosylase *TOUCH4* (At5g57560) gene expression after treatment with auxin, $_{His}$AtRALF1 and brassinolide. Total RNA was extracted from roots of 10-d-old wild type plants 5, 30, 60 and 120 min after treatment with 1 µM of the auxin indole-3-acetic acid (IAA), 1 µM of $_{His}$AtRALF1 (R) or 1 µM of brassinolide (BL). *GAPDH* expression was used as a reference gene. C, control plants treated with water. See Supplementary Table S2 for ratios of the band intensities.

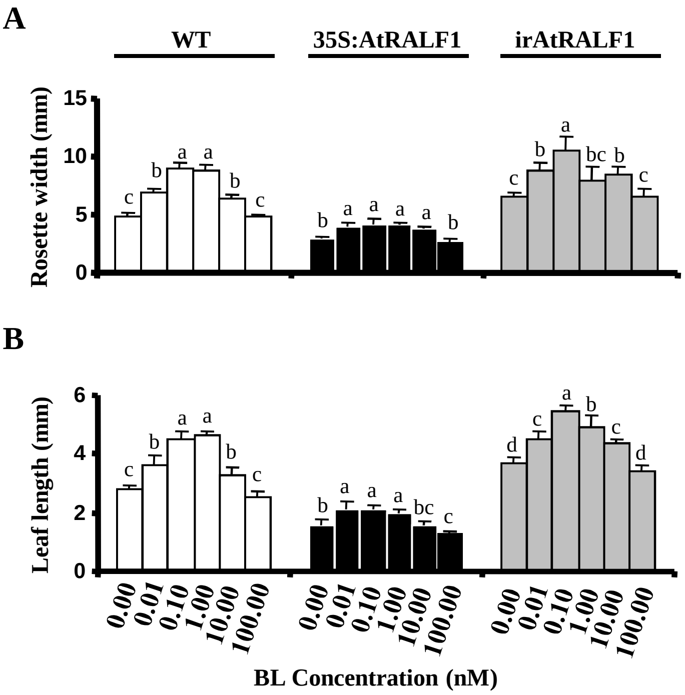

Supplementary Fig. S6. AtRALF1-overexpressing (35S:AtRALF1, black columns) and AtRALF1-silencing (irAtRALF1, gray columns) transgenic lines treated with different concentrations of brassinolide (BL). (A) Rosette length. (B) Length of the 7th leaf. Rosettes and leaves were measured in 5-d-old seedlings (n>30) grown in the presence or absence (control plants, white columns) of BL. Error bars indicate SD. Statistical analysis was made for each genetic background separately. Columns within each genotype that are followed by the same letter are not significantly different (*p-value* <0.01). The experiment was performed at least three times.

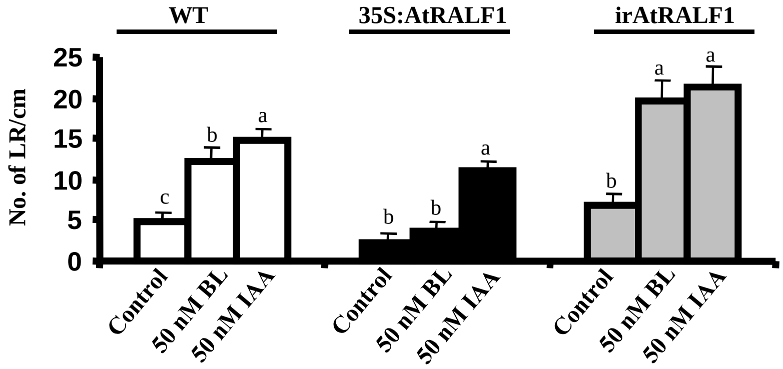

Supplementary Fig. S7. Number of emerged lateral roots in AtRALF1-overexpressing (35S:AtRALF1, black columns) and AtRALF1-silencing (irAtRALF1, gray columns) transgenic lines treated with brassinolide (BL) or indole-3-acetic acid (IAA). The number of emerged lateral roots was measured in 10-d-old seedlings (n>30) grown in the presence or absence (control plants, white columns) of BL or IAA. Error bars indicate SD. Statistical analysis was made for each genetic background separately. Columns within each genotype that are followed by the same letter are not significantly different (*p-value* <0.01). The experiment was performed at least three times.

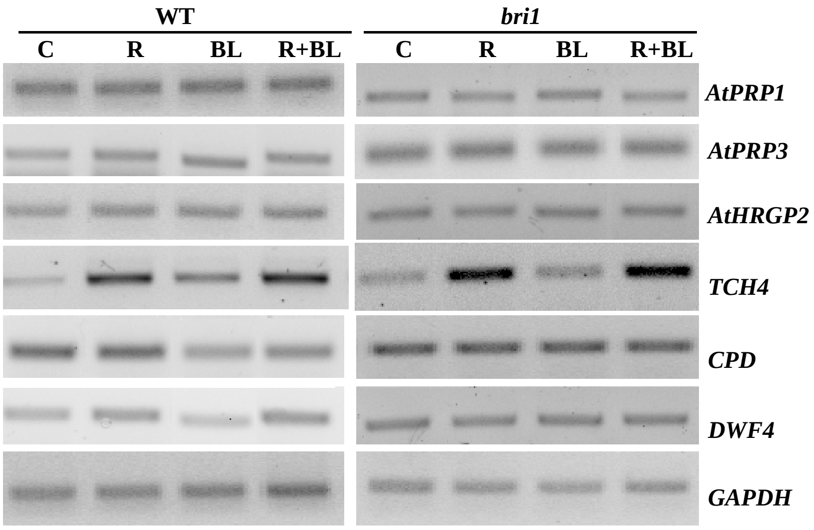

Supplementary Fig. S8. Gene expression analyses of AtRALF1-inducible genes upon simultaneous treatment with $_{His}$AtRALF1 (R, 1 μM for 30 min) and brassinolide (BL, 1 μM for 30 min). Semi-quantitative RT-PCR performed using total RNA extracted from roots of untreated (C) or treated (R, BL or R+BL) 10-d-old wild type (WT) or brassinolide insensitive mutant *bri1* plants. *GAPDH* expression was used as a control. *AtPRP1* and *AtPRP3*, proline-rich proteins 1 and 3 (*AtPRP1*, At1g54970 and *AtPRP3*, At3g62680). *AtHRGP2*, hydroxyproline-rich glycoprotein (At5g19800). *TCH4*, xyloglucan endotransglucosylase TOUCH4 (At5g57560). *CPD*, constitutive photomorphism and dwarfism (At5g05690). *DWF4*, DWARF4 (At3g50660). *GAPDH*, glyceraldehyde-3-phosphate dehydrogenase (At1g13440). The experiment was performed at least three times (independent biological replicates). See Supplementary Table S2 for ratios of the band intensities.

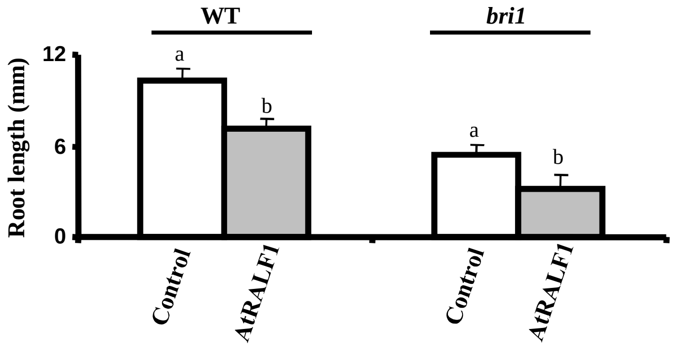

Supplementary Fig. S9. $_{His}$AtRALF1 effect on root length in *bri1* mutants. Root length of light-grown 5-d-old seedlings $_{His}$AtRALF1-treated (1 µM, gray columns) or untreated (control, white columns). Error bars indicate SD. Statistical analysis was made for each genetic background separately. Columns within each genotype that are followed by the same letter are not significantly different (*p-value* < 0.01). The experiment was performed at least three times.

Supplementary Table S1. Primers used for cloning, quantitative and semi-quantitative RT-PCR analysis.

Gene (locus)	Primer name	Sequence (5' – 3')
At1g02900	At1g02	FW: TGTAGCATGGGCTACGACTG<br>RV: AAGGCACACTGTTCCGTTTC
At1g02900	AtRALF1	FW: CACCATGGACAAGTCCTTTACTCTGT<br>RV: ACTCCTGCAAGCAGCAATTT
At1g02900	A	TCCATGGCGACCACCAAATACATTAGCTATCAGTCTTTGAAA<br>CGTAACAGTGTGCCGTGTTACCGCCGCGGTGCGTCTT
At1g02900	B	ATAAGCTTTTAACTGCGGCAACGAGCAA
At1g02900	C	TTCTCGAGTGCACTCCTGCAACGAGCAATT
At1g02900	D	TTCATATGGCGACCACAAAATACATAAGCT
At1g54970	PRP1	FW: AAGATTGTGTGCTCGGAGAGG<br>RV: CCCTTGTTGACATTGGTTGG
At3g62680	PRP3	FW: GTTCCGACCCAGCATCATAC<br>RV: GCAAGTCTCGACCGGAGATA
At5g19800	HRGP2	FW: CACAGTTGCTAATGGAAACGA<br>RV: CGGCGGTGAATATCTCTTGT
At5g05690	CPD	FW: AGCAACTCGGTAACGACAGG<br>RV: CAGAGAGTGCAACCCTAGCC
At3g50660	DWF4	FW: GTGGGTGGAAAGTGTTACCG<br>RV: CTGTTGCCATCTCCAAGGAT
At5g57560	TCH4	FW: TCCTAATGCCTCGAAACAGG<br>RV: CTGCACCCATCTCATCCTTT
At2g33775	AtRALF19	FW: CACCGCCGCGAGGCGCAGCTACATCAGTTAC<br>RV: CCCTCGAGTTAAGAAGTTTGCCTGTAGCA
At3g05490	AtRALF22	FW: CACCGCACAGAAGAAGTACATTAGCTACGGTGCTATGCGTCGTAACAGCGTGCCGTTGCAG<br>RV: TCAACGACGGCAACGAGTGATGGTGCTGCAGCCACGGCTGTACGGATTCGCCTGAGCGCC
At3g16570	AtRALF23	FW: CACCGCTACGAGGAGGTACATC<br>RV: AAAAGCTTTCATGAGCGCCGGCAGCGAGTATC
At3g23805	AtRALF24	FW: CACCATGATGCGGAAACAGTACATA<br>RV: CCAAGCTTTCAAGTCTTGATGTCGTT
At4g13950	AtRALF31	FW: CACCGCGCAGAAACGGTACATC<br>RV: AACTCGAGTCATGTGTTGATGTCGTTTGT
At4g15800	AtRALF33	FW: GGGAATTCCGCAACAACGAAGTATATA<br>RV: AAAAGCTTTTATCGCCTGCAACGAGT
At5g67070	AtRALF34	FW: TTCATATGTACTGGCGGAGGACGAAGTA<br>RV: TAAGCTTCTAGCGGCGGCAGCGGGTGATCG
At1g13440	GAPDH	FW: TTGGTGACAACAGGTCAAGCA<br>RV: AAACTTGTCGCTCAATGCAA

Supplementary Table S2. Evaluation of RT-PCR signals via densitometry after normalization to the housekeeping gene *GAPDH* (glyceraldehyde-3-phosphate dehydrogenase, At1g13440).

Gene	Ratio				
Fig. 1A					
	35S:AtRALF1/WT	irAtRALF1-1/WT	irAtRALF1-19/WT	irAtRALF1-23/WT	
AtRALF1	8.14	0.21	0.90	0.71	
Fig. 3A					
	35S:AtRALF1/WT	irAtRALF1/WT			
AtPRP1	4.11	0.03			
AtPRP3	2.74	0.97			
AtHRGP2	3.14	0.78			
TCH4	4.41	0.01			
Fig. 3C					
	0.01/0 µM BL	0.1/0 µM BL	0.5/0 µM BL	1/0 µM BL	5/0 µM BL
AtPRP1	0.97	1.31	1.69	2.01	2.12
AtPRP3	1.13	1.43	1.49	1.98	2.05
AtHRGP2	3.33	6.05	6.45	8.05	8.16
TCH4	0.97	1.39	1.48	1.89	2.00
Fig. 6A					
	35S:AtRALF1/WT	irAtRALF1/WT			
CPD	1.96	0.47			
DWF4	2.27	0.87			
Fig. 6C					
	0.01/0 µM BL	0.1/0 µM BL	0.5/0 µM BL	1/0 µM BL	5/0 µM BL
CPD	1.23	1.33	1.67	2.50	2.65
DWF4	0.99	1.33	1.39	1.94	2.01
Fig. 7A					
	WT background				
	$_{His}$AtRALF1/untreated	BL/untreated	$_{His}$AtRALF1 + BL/untreated		
AtPRP1	6.77	1.09	6.30		
AtPRP3	3.25	1.02	2.68		
AtHRGP2	2.58	0.94	1.44		
TCH4	2.35	1.29	1.30		
CPD	2.60	0.66	2.23		
DWF4	5.43	0.02	2.09		
	bri1 background				
	$_{His}$AtRALF1/untreated	BL/untreated	$_{His}$AtRALF1 + BL/untreated		
AtPRP1	6.95	1.00	6.96		
AtPRP3	3.35	1.07	3.37		
AtHRGP2	3.40	0.97	3.35		
TCH4	5.03	0.93	4.98		
CPD	1.85	1.01	2.00		
DWF4	5.40	0.93	5.27		
Fig. S3					
	irAtRALF1/WT				
AtRALF1	0.23				
AtRALF19	1.13				
AtRALF22	1.02				
AtRALF23	1.07				
AtRALF24	0.90				
AtRALF31	1.03				
AtRALF33	1.10				
AtRALF34	1.13				
Fig. S4					
	$_{His}$AtRALF1/untreated				
AtPRP1	1.01				
AtPRP3	0.99				
AtHRGP2	0.95				
TCH4	1.43				
CPD	1.01				
DWF4	0.92				
Fig. S5					
	5 min treatment				
	IAA/untreated	BL/untreated	$_{His}$AtRALF1/untreated		
TCH4	2.92	0.99	5.22		

Table continues on following page.

Supplementary Table S2. *(Continued from previous page.)*

	Gene	Ratio		
Fig. S5				
		30 min treatment		
		IAA/No-treated	BL/untreated	$_{His}$AtRALF1/untreated
	TCH4	2.89	1.56	4.85
		60 min treatment		
		IAA/No-treated	BL/untreated	$_{His}$AtRALF1/untreated
	TCH4	1.32	1.62	4.19
		120 min treatment		
		IAA/untreated	BL/untreated	$_{His}$AtRALF1/untreated
	TCH4	1.17	4.24	3.90
Fig. S8				
		WT background		
		$_{His}$AtRALF1/untreated	BL/untreated	$_{His}$AtRALF1 + BL/untreated
	AtPRP1	1.08	1.03	1.07
	AtPRP3	1.16	1.06	1.10
	AtHRGP2	1.06	1.01	1.03
	TCH4	1.47	1.25	1.40
	CPD	1.08	0.51	0.83
	DWF4	1.19	0.71	1.07
		bri1 background		
		$_{His}$AtRALF1/untreated	BL/untreated	$_{His}$AtRALF1 + BL/untreated
	AtPRP1	1.02	1.06	1.01
	AtPRP3	1.15	1.05	1.12
	AtHRGP2	0.97	1.03	1.05
	TCH4	1.32	0.97	1.31
	CPD	1.01	1.07	1.03
	DWF4	0.99	1.01	1.08
